# Prognostic evaluation of the norepinephrine equivalent score and the vasoactive-inotropic score in patients with sepsis and septic shock: a retrospective cohort study

**DOI:** 10.3389/fcvm.2024.1415769

**Published:** 2024-08-02

**Authors:** Wenzhe Li, Yi Wang, Buzukela Abuduaini, Xiang Li, Pengfei Pan, Jian Cui, Xiangyou Yu

**Affiliations:** ^1^ Department of Critical Care Medicine, The First Affiliated Hospital of Xinjiang Medical University, Urumqi, Xinjiang, China; ^2^ Xinjiang Key Laboratory of Medical Animal Model Research, Urumqi, Xinjiang, China; ^3^ Department of Critical Care Medicine, Chongqing University Three Gorges Hospital, Chongqing, China

**Keywords:** sepsis, vasoactive agents, vasoactive-inotropic score, norepinephrine equivalent score, 28-day mortality

## Abstract

**Background:**

This study investigated the association between vasoactive medication exposure and mortality risk in patients with sepsis using the norepinephrine equivalent (NEE) score and vasoactive-inotropic score (VIS).

**Methods:**

This retrospective cohort study included adult patients with sepsis requiring vasoactive agents. The data were extracted from the Medical Information Mart for Intensive Care IV database. The primary outcome was 28-day mortality. Multivariate Cox regression was used to elucidate the relationship between vasoactive medication exposure and 28-day mortality, as quantified by the VIS and NEE score. Hazard ratios with 95% confidence intervals (CI) for 28-day mortality were generated, and forest plots were constructed to present the results of univariate and multivariate analyses. The Kaplan–Meier method was used to analyze the cumulative incidence of 28-day mortality. A nomogram was constructed to predict the prognosis of patients with sepsis.

**Results:**

The present study encompassed 9,032 patients diagnosed with sepsis who received vasoactive therapy, of which 4,229 patients were further analyzed at the second hour after the onset of sepsis. Distinct variations in demographic data were observed between survivors (*n* = 3,265, 77.21%) and non-survivors (*n* = 964, 22.79%). Multivariate analysis indicated that several factors, including VIS >15.04 (*p* = 0.001), NEE >0.10 (*p* < 0.001), heart rate (*p* = 0.045), mean arterial pressure (*p* = 0.009), respiratory rate (*p* < 0.001), oxygen saturation (*p* < 0.001), blood urea nitrogen (BUN) (*p* = 0.001), and the Acute Physiology and Chronic Health Evaluation II (*p* < 0.001), were significantly associated with 28-day mortality in the patients with sepsis. The NEE score, respiratory rate, oxygen saturation, and BUN were incorporated into the nomogram model with a concordance index of 0.779 and an area under the curve of 0.802 (95% CI 0.787–0.818).

**Conclusion:**

We found that the VIS and NEE score had favorable values for predicting mortality risk in patients with sepsis in the intensive care units. The VIS and NEE score in the second hour after sepsis onset were independently associated with 28-day mortality in patients with sepsis.

## 1 Introduction

Numerous studies have defined sepsis as a life-threatening organ dysfunction caused by a dysregulated host response to infection (1). Although various interventions to improve prognosis have been tested, sepsis remains a predominant global burden with a high prevalence and mortality rate (2–4). Distributive shock is the most common form of circulatory shock encountered in patients with sepsis, and effective hemodynamic support is fundamental for protecting and restoring organ dysfunction, and adequate mean arterial pressure (MAP) is widely considered a primary necessity for maintaining organ perfusion (5, 6). Besides fluid resuscitation, vasoactive medications have been routinely administered for decades in patients with septic shock. Guidelines recommend using norepinephrine as the first-line agent and adding vasopressin and epinephrine instead of increasing the norepinephrine dose (7, 8). Although these agents can partially alter the trajectory of hemodynamic instability and early vasopressor initiation, which are independently associated with decreased mortality risk, their safety has not been formally tested (9, 10). Consequently, along with growing concerns about the catecholamine burden, rationalizing an early multimodal balanced vasopressor strategy as an alternative to the classic stepwise approach seems reasonable. However, the use of high-dose vasoactive drugs may lead to side effects such as increased myocardial oxygen consumption, arrhythmias, decreased microcirculatory perfusion, and even organ ischemia. There is still considerable heterogeneity in using vasoactive agents in clinical practice (11, 12). The cumulative dose of vasoactive medication may be an easily identifiable objective measure for predicting the prognosis of patients with sepsis and septic shock. The vasoactive-inotropic score (VIS) and norepinephrine equivalent (NEE) score quantify the total number of inotropes and/or vasopressors and can objectively provide an index of the degree of hemodynamic support (13, 14). We hypothesized that high-dose vasoactive agent administration is associated with an increased risk of death in critically ill patients with sepsis. This trial was conducted to determine the association between vasoactive medication exposure and mortality risk in patients with sepsis using the VIS and NEE score.

## 2 Methods

### 2.1 Sources of data

This retrospective study was conducted using the Medical Information Mart for Intensive Care (MIMIC-IV) database (15). The database contains records of 73,181 hospital admissions to intensive care units (ICUs) at the Beth Israel Deaconess Medical Center between 2008 and 2019. Two authors extracted the data from the database (WZL no. 57264471 and XL no. 46830776). The protocol, analysis, and findings are reported in a standardized format recommended by the Strengthening the Reporting of Observational Studies in Epidemiology (STROBE) statement.

### 2.2 Population selection criteria

Adult patients who were critically ill were eligible for enrollment if their condition fulfilled the Third International Consensus Definitions for Sepsis and Septic Shock (Sepsis-3) (1). Sepsis was defined as a suspected or confirmed infection when the Sequential Organ Failure Assessment (SOFA) score increased 2 points or more. The following exclusion criteria were used: (1) patients who did not meet the diagnostic criteria for sepsis and (2) patients who died before the initiation of vasoactive medication administration. In addition, only the first admission records of the first ICU admission of the first hospital stay were analyzed.

### 2.3 Data collection and definitions

Data were extracted using PostgreSQL 15 (https://www.postgresql.org/download/) and Navicat Premium 16.1.10 (https://www.navicat.com/en/download/navicat-premium). The patients’ baseline characteristics, including age, sex, body weight, height, the Charlson comorbidity index (CCI), and coexisting diseases, were extracted. Initial vital signs of sepsis onset (heart rate, MAP, respiratory rate, oxygen saturation, and body temperature) and laboratory test results [white blood cells (WBC), platelets, hemoglobin, hematocrit, blood urea nitrogen (BUN), and serum creatinine] were collected. Scores for assessing illness severity, including the Acute Physiology and Chronic Health Evaluation II (APACHE II) and SOFA scores, were also extracted.

Complete vasoactive medication administration records were screened for analysis. The VIS was defined as the formula for calculating vasoactive agents and inotropes based on dopamine dose (µg/kg/min) (13). The NEE score is defined as a formula for calculating vasoactive agents with norepinephrine dose (µg/kg/min) as a benchmark, excluding inotropes (14). There are different weight assignments in the formulas for the VIS and NEE score.

$$\begin{array}{c} \mathrm{VIS}=\mathrm{dopamine}\;\mathrm{dose}\;(\mu g/\mathrm{kg}/\min)+\mathrm{dobutamine}\;\mathrm{dose}\;\;(\mu g/\mathrm{kg}/\min)+100\times \\ \mathrm{epinephrine}\;\mathrm{dose}\;\;(\mu g/\mathrm{kg}/\min)+100\times \mathrm{norepinephrine}\;\mathrm{dose}(\mu g/\mathrm{kg}/\min)+\;\\ 10,000\times \mathrm{vasopressin}\;\mathrm{dose}(U/\mathrm{kg}/\min)+10\times \mathrm{milrinone}\;\mathrm{dose}\;(\mu g/\mathrm{kg}/\min)+\;\\ \begin{array}{c} \mathrm{enoximone}\;\mathrm{dose}\;(\mu g/\mathrm{kg}/\min)+50\times \mathrm{levosimendan}\;\mathrm{dose}\;(\mu g/\mathrm{kg}/\min)+25\times \\ \mathrm{olprinone}\;\mathrm{dose}\;(\mu g/\mathrm{kg}/\min)+20\times \mathrm{methylene}\;\mathrm{blue}\;\mathrm{dose}\;(\mathrm{mg}/\mathrm{kg}/h)+10\times \\ \begin{array}{c} \mathrm{phenylephrine}\;\mathrm{dose}\;(\mu g/\mathrm{kg}/\min)+10\times \mathrm{terlipressin}\;\mathrm{dose}\;(\mu g/\min)+0.25\times \\ \mathrm{angiotensin}\;\mathrm{II}\;\mathrm{dose}(\mathrm{ng}/\mathrm{kg}/\min)\; \end{array} \end{array} \end{array}$$

$$\begin{array}{c} \mathrm{NEE}=\mathrm{norepinephrine}\;\mathrm{dose}\;(\mu g/\mathrm{kg}/\min)+\mathrm{epinephrine}\;\mathrm{dose}\;\;(\mu g/\mathrm{kg}/\min)+\\ 1/100\times \mathrm{dopamine}\;\mathrm{dose}\;\;(\mu g/\mathrm{kg}/\min)+0.06\times \mathrm{phenylephrine}\;\mathrm{dose}(\mu g/\mathrm{kg}/\min)+\;\\ 2.5\times \mathrm{vasopressin}\;\mathrm{dose}(U/\min)+0.0025\times \mathrm{angiotensin}\;\mathrm{II}\;\mathrm{dose}\;(\mathrm{ng}/\mathrm{kg}/\min)+10\;\\ \begin{array}{c} \times \;\mathrm{terlipressin}\;\mathrm{dose}\;(\mu g/\mathrm{kg}/\min)+0.2\times \mathrm{methylene}\;\mathrm{blue}\;\mathrm{dose}\;(\mathrm{mg}/\mathrm{kg}/h)+8\times \\ \mathrm{metaraminol}\;\mathrm{dose}\;(\mu g/\mathrm{kg}/\min)+0.02\times \mathrm{hydroxocobalamin}\;\mathrm{dose}\;(g)+0.4\times \\ \mathrm{midodrine}\;\mathrm{dose}\;(\mu g/\mathrm{kg}/\min) \end{array} \end{array}$$

### 2.4 Outcomes

The main outcome was death from any cause, 28 days after the diagnosis of sepsis. The key secondary outcomes included death in the ICU at 7 and 14 days, or during hospitalization. The patient outcomes were extracted from the database and defined computationally by recording the time nodes.

### 2.5 Statistical analysis

The predictive values of the VIS and NEE score for mortality at each hour were assessed using the receiver operating characteristic (ROC) curve and area under the curve (AUC). The most suitable cutoff values were summarized hourly on the first day after the onset of sepsis. Baseline data are presented based on different types of variables. The Shapiro–Wilk test was used to test the normality of the variables. Continuous variables are presented as mean [standard deviation (SD)] and median [interquartile range (IQR 25%–75%)] for normally and non-normally distributed data, respectively. Categorical variables are presented as numbers (percentages). The chi-squared test, t-test, or Mann–Whitney U-test was used to compare patients’ baseline characteristics between the survival and non-survival groups, as appropriate. Univariate Cox regression analysis was conducted to determine the association between variables with predictive value and mortality risk. Variables with a *p*-value <0.05 in univariate analysis were selected for further multivariate Cox regression analysis. Hazard ratio (HR) with a 95% confidence interval (CI) for 28-day mortality was generated using multivariate analysis. Forest plots were generated to present the results of the univariate and multivariate analyses. The Kaplan–Meier method was used to analyze the cumulative incidence of 28-day mortality. A nomogram was constructed and complex regression equations were transformed into visual graphs. Calibration plots and the ROC were used to determine the predictive accuracy of the nomogram model. R software (version 4.2.3; R Foundation for Statistical Computing, Vienna, Austria) was used for the statistical analyses. Statistical significance was set at *p* <0.05.

## 3 Results

### 3.1 Baseline characteristics

**Figure 1:**
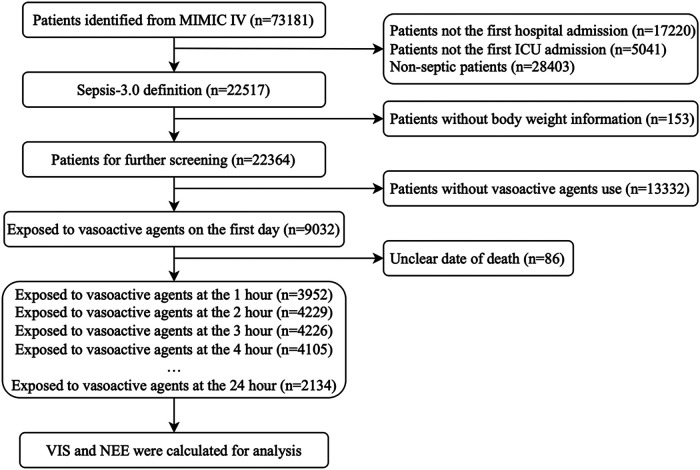
The flow diagram of study patients.

**Figure 2:**
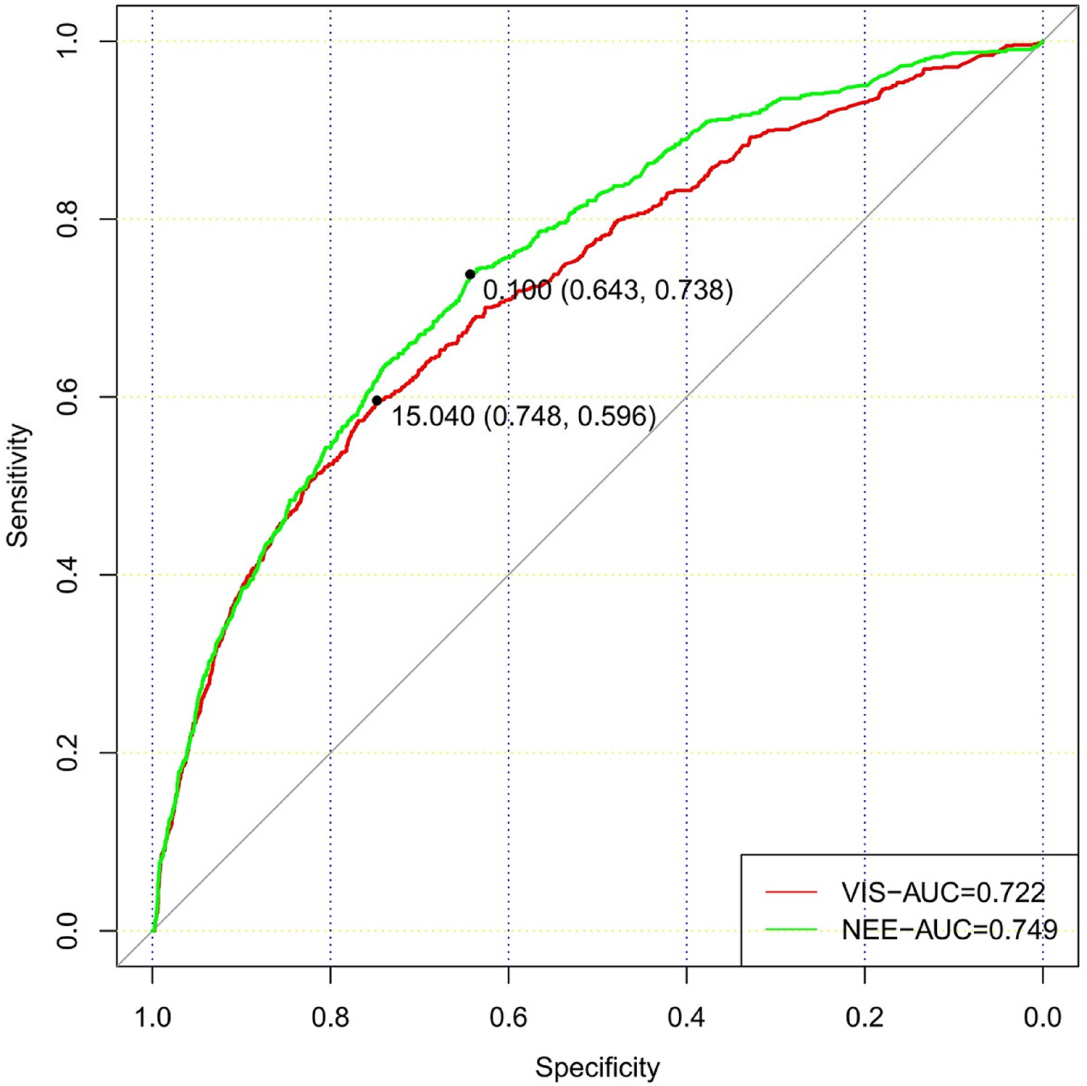
Comparison of the ability to predict 28-day mortality between the VIS and NEE score in the second hour.

**Figure 3:**
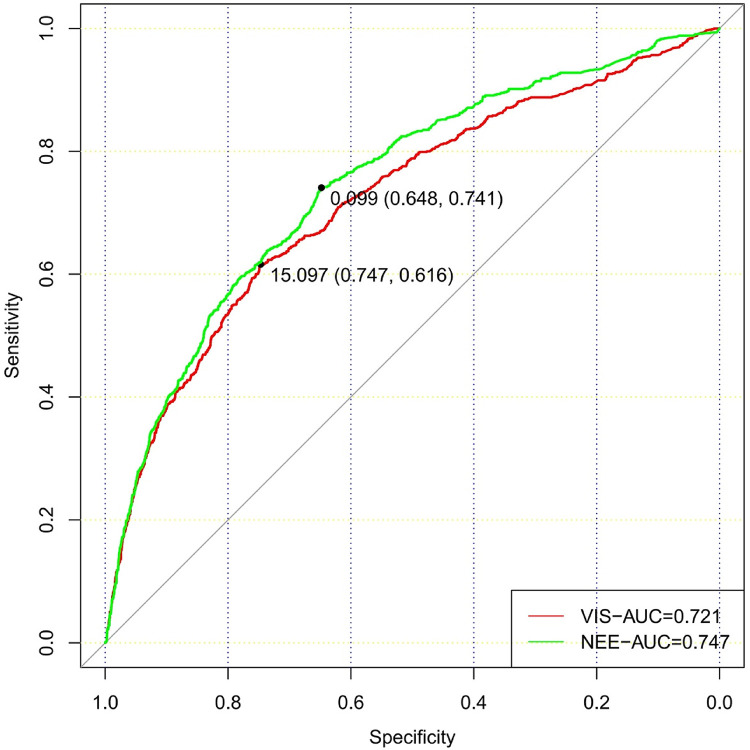
Comparison of the ability to predict 28-day mortality between the VIS and NEE score in the fourth hour.

In total, 73,181 patients in the database were screened for enrollment, and 9,032 patients exposed to vasoactive agents were included in the analysis (Figure 1). The study cohort calculated the VIS and NEE score each hour after sepsis diagnosis. As the first step, the VIS and NEE score correlations with the outcomes were calculated, and ROC curve analysis to predict mortality using each VIS and NEE score showed different AUCs, sensitivities, and specificities (Supplementary Tables S1–S3). A comparison of the ability to predict the 28-day mortality between the VIS and NEE score groups is outlined in Figures 2, 3. At the second hour, the AUC, sensitivity, and specificity for VIS = 15.040 were 72.2%, 74.8%, and 59.6%, respectively. The AUC, sensitivity, and specificity values for NEE score = 0.100 were 74.9%, 64.3%, and 73.8%, respectively (Figure 2). At the fourth hour, the AUC, sensitivity, and specificity values for VIS = 15.097 were 72.1%, 74.7%, and 61.6%, respectively. The AUC, sensitivity, and specificity for NEE score = 0.099 were 74.7%, 64.8%, and 74.1%, respectively (Figure 3). The VIS and NEE score measured at the second hour had the best predictive value for 7-, 14-, and 28-day mortality according to the AUC (Supplementary Figures S1–S4), and 4,229 patients at this time point were included in further analysis. Demographic data comparing survivors (*n* = 3,265, 77.21%) and non-survivors are outlined in Table 1. The mortality rates at 7 and 14 days were 15.23% (*n* = 644) and 19.34% (*n* = 818), respectively. The 28-day mortality was 22.79% (*n* = 964).

**Table 1:** Demographic data and baseline characteristics.

Variables	All patients (*n* = 4,229)	Survivors (*n* = 3,265)	Non-survivors (*n* = 964)	*p*-value
Age (years), mean (SD)	67.7 (14.4)	66.9 (14.1)	70.3 (15.2)	<0.001
Gender (male), *n* (%)	1,639 (38.8%)	1,205 (36.9%)	434 (45.0%)	<0.001
Height (cm), mean (SD)	170 (10.2)	170 (10.0)	168 (10.4)	<0.001
Charlson comorbidity index, mean (SD)	4.99 (2.74)	4.64 (2.58)	6.18 (2.94)	<0.001
SOFA, mean (SD)	4.37 (2.26)	4.11 (2.02)	5.25 (2.76)	<0.001
APACHE II, mean (SD)	21.5 (7.69)	19.9 (6.82)	26.9 (7.99)	<0.001
Comorbidities, *n* (%)				
Liver disease	614 (14.5%)	354 (10.8%)	260 (26.9%)	<0.001
Hypertension	2,778 (65.7%)	2,192 (67.2%)	586 (60.7%)	<0.001
Cerebrovascular disease	453 (10.7%)	310 (9.5%)	143 (14.8%)	<0.001
Chronic pulmonary disease	1,081 (25.6%)	808 (24.8%)	273 (28.3%)	0.03
Congestive heart failure	1,274 (30.1%)	922 (28.2%)	352 (36.5%)	<0.001
Peripheral vascular disease	599 (14.2%)	458 (14.0%)	141 (14.6%)	0.688
Renal disease	831 (19.7%)	580 (17.8%)	251 (26.0%)	<0.001
Metastatic solid tumor	202 (4.8%)	105 (3.2%)	97 (10.1%)	<0.001
Hematologic malignancy	485 (11.5%)	317 (9.7%)	168 (17.4%)	<0.001
Vital signs, mean (SD)				
Heart rate (beats/min)	71.1 (15.1)	70.4 (13.8)	73.4 (18.6)	<0.001
Mean arterial pressure (mmHg)	73.4 (7.41)	73.7 (6.67)	72.3 (9.42)	<0.001
Respiratory rate (breaths/min)	19.7 (4.17)	18.9 (3.73)	22.1 (4.62)	<0.001
Body temperature (°C)	36.8 (0.70)	36.9 (0.59)	36.6 (0.97)	<0.001
Oxygen saturation (%)	97.1 (3.13)	97.5 (1.78)	95.5 (5.38)	<0.001
Laboratory tests, mean (SD)				
WBC (10^9^/L)	17.8 (10.9)	17.4 (9.24)	19.3 (15.1)	<0.001
Hematocrit (%)	28.6 (6.02)	28.3 (5.61)	29.4 (7.17)	<0.001
Hemoglobin (g/dl)	9.50 (1.97)	9.49 (1.84)	9.54 (2.35)	0.538
Platelet (10^9^/L)	160 (89.7)	160 (84.0)	160 (107)	0.866
BUN (mg/dl)	31.1 (24.2)	27.2 (20.8)	44.4 (29.4)	<0.001
Creatinine (mg/dl)	1.75 (1.59)	1.56 (1.49)	2.39 (1.73)	<0.001
Vasopressors use in the second hour, median (IQR)				
Norepinephrine dose (µg/kg/min)	0.140 (0.209)	0.100 (0.140)	0.200 (0.281)	0.204
Epinephrine dose (µg/kg/min)	0.040 (0.060)	0.030 (0.030)	0.103 (0.151)	<0.001
Dopamine dose (µg/kg/min)	8.04 (10.00)	7.51 (5.22)	10.0 (14.20)	0.021
Phenylephrine dose (µg/kg/min)	0.800 (1.00)	0.700 (0.502)	2.00 (3.000)	<0.001
Vasopressin dose (U/min)	2.40 (0.007)	2.40 (0.014)	2.40 (0.005)	0.213
Angiotensin II dose (ng/kg/min)	0.020 (0.001)		0.020 (0.001)	
Dobutamine (µg/kg/min)	5.00 (4.26)	5.00 (2.51)	5.01 (5.38)	0.094
Milrinone (µg/kg/min)	0.800 (1.00)	0.700 (0.502)	2.00 (3.00)	0.295
VIS and NEE in the second hour, median (IQR)				
VIS	10.0 (15.0)	9.01 (10.3)	20.0 (32.0)	<0.001
NEE score	0.080 (0.160)	0.060 (0.107)	0.200 (0.311)	<0.001

Overall, a higher age [70.3 (15.2) vs. 66.9 (14.1); *p* < 0.001], Charlson comorbidity index [6.18 (2.94) vs. 4.64 (2.58); *p* < 0.001], SOFA score [5.25 (2.76) vs. 4.11 (2.02); *p* < 0.001], and APACHE II score [26.9 (7.99) vs. 19.9 (6.82); *p* < 0.001] were observed in the non-survival group patients. There were more comorbidities in the non-survivor group. In addition, the vital signs also differed between the two groups. A more rapid respiratory rate [22.1 (4.62) vs. 18.9 (3.73); *p* < 0.001] and lower oxygen saturation [95.5 (5.38) vs. 97.5 (1.78); *p* < 0.001] were manifested in non-survival patients. Furthermore, higher levels of WBC, BUN, and serum creatinine were observed in non-survivors (Table 1).

### 3.2 Vasoactive medications and scores

Patients received norepinephrine, epinephrine, dopamine, phenylephrine, dobutamine, milrinone, vasopressin, and angiotensin II; the median doses were 0.140 (0.209) µg/kg/min in 2,054 (48.57%) patients, 0.040 (0.060) µg/kg/min in 330 (7.80%) patients, 8.04 (10.0) µg/kg/min in 204 (4.82%) patients, 0.800 (1.00) µg/kg/min in 2,125 (50.25%) patients, 5.00 (4.26) µg/kg/min in 86 (2.03%) patients, 0.800 (1.00) µg/kg/min in 106 (2.51%) patients, 2.40 (0.007) U/min in 562 (13.29%) patients, and 0.020 (0.001) ng/kg/min in 4 (0.09%) patients. None of the patients received other drugs in the VIS or NEE score formulas. The VIS of the non-survivor group was much higher [20.0 (32.0) vs. 9.01 (10.3); *p* < 0.001] than those of the non-survivor and NEE score groups [0.200 (0.311) vs. 0.060 (0.107); *p* < 0.001] (Table 1).

### 3.3 Cox regression analyses of predictors for 28-day mortality

**Figure 4:**
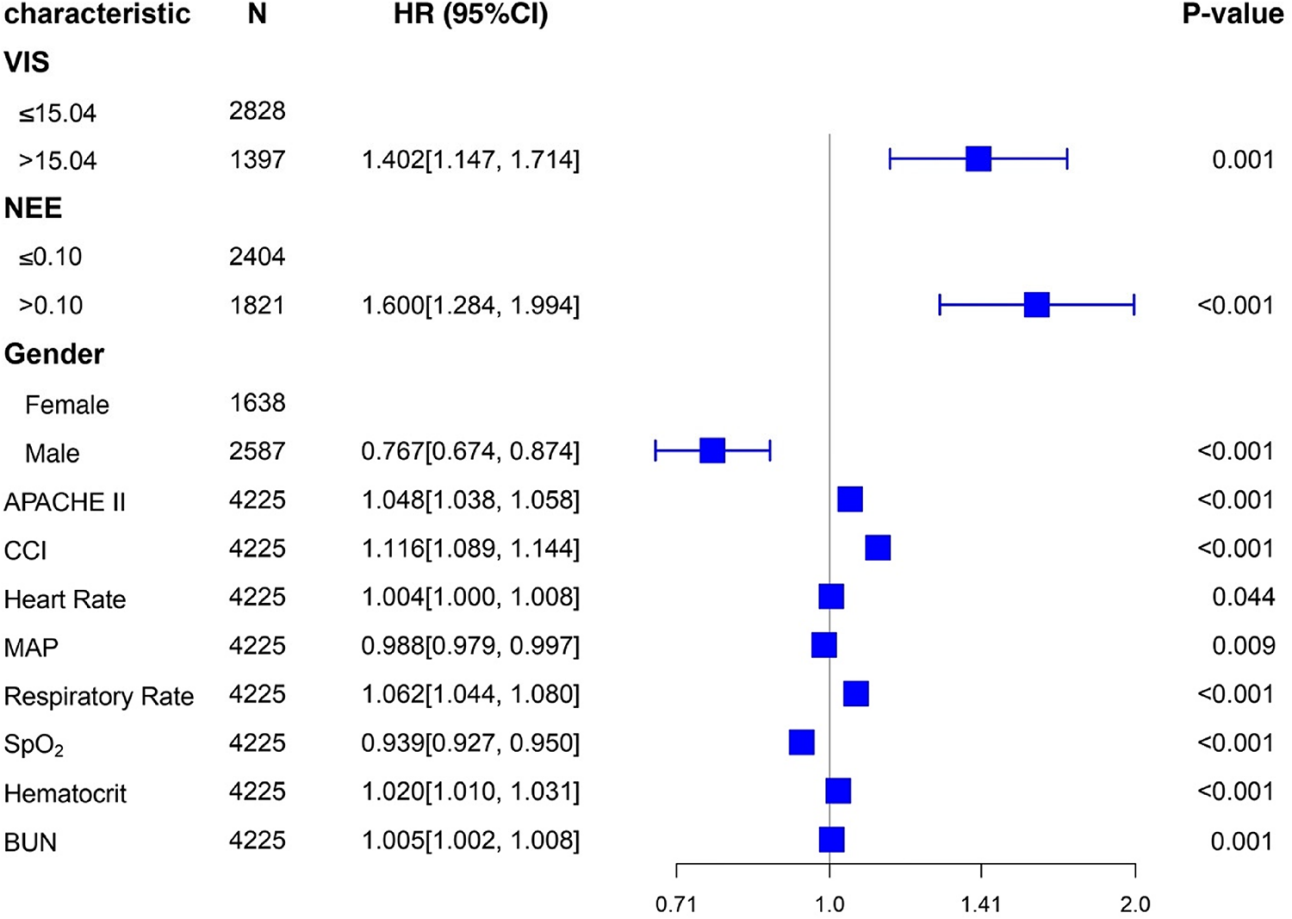
Forest plots of multivariable hazard ratios for the primary endpoint in different variables.

Table 2 summarizes the predictive values of the variables for 28-day mortality in patients evaluated using univariate and multivariate Cox regression analyses. Forest plots presenting the results of the Cox regression analyses demonstrated a relationship between different variables and the risk of death within 28 days in the patients (Figure 4). In the univariate Cox proportional hazard model, VIS >15.04, NEE score >0.10, sex, age >65 years, SOFA score, APACHE II score, the Charlson comorbidity index, heart rate, MAP, respiratory rate, oxygen saturation, WBC count, hematocrit, BUN, and creatinine were associated with the primary outcome (*p* < 0.001) (Supplementary Figure S5). Parameters with significant results in univariate analysis were evaluated using multivariate Cox regression analysis. VIS (*p* = 0.001), NEE score (*p* < 0.001), gender (*p* < 0.001), APACHE II score (*p* < 0.001), the Charlson comorbidity index (*p* < 0.001), HR (*p* = 0.044), MAP (*p* = 0.009), respiratory rate (*p* < 0.001), oxygen saturation (*p* < 0.001), hematocrit (*p* < 0.001), and BUN (*p* = 0.001) were associated with 28-day mortality in study patients according to the multivariate analysis (Table 2).

**Table 2:** Predictors of 28-day mortality using the Cox proportional hazard model.

Characteristic	*N*	Univariable HR (95% CI)	*p*-value	Multivariable HR (95% CI)	*p*-value
VIS					
≤15.04	2,828	1 (reference group)		1 (reference group)	
>15.04	1,397	3.700 (3.255–4.212)	<0.001	1.402 (1.147–1.714)	0.001
NEE score					
≤0.10	2,404	1 (Reference group)		1 (Reference group)	
>0.10	1,821	4.020 (3.493–4.621)	<0.001	1.600 (1.284–1.994)	<0.001
Gender					
Female	1,638	1 (Reference group)		1 (Reference group)	
Male	2,587	0.750 (0.660–0.851)	<0.001	0.767 (0.674–0.874)	<0.001
Age					
≤65	1,659	1 (Reference group)		1 (Reference group)	
>65	2,566	1.290 (1.131–1.477)	<0.001	1.119 (0.960–1.305)	0.150
SOFA	4,225	1.180 (1.158–1.212)	<0.001	1.018 (0.991–1.046)	0.184
APACHE II	4,225	1.100 (1.091–1.106)	<0.001	1.048 (1.038–1.058)	<0.001
CCI	4,225	1.180 (1.151–1.200)	<0.001	1.116 (1.089–1.144)	<0.001
Heart rate	4,225	1.020 (1.020–1.028)	<0.001	1.004 (1.000–1.008)	0.044
MAP	4,225	0.970 (0.963–0.982)	<0.001	0.988 (0.979–0.997)	0.009
Respiratory rate	4,225	1.150 (1.138–1.166)	<0.001	1.062 (1.044–1.080)	<0.001
SpO_2_	4,225	0.890 (0.882–0.897)	<0.001	0.939 (0.927–0.950)	<0.001
WBC	4,225	1.010 (1.008–1.017)	<0.001	0.998 (0.992–1.003)	0.440
Hematocrit	4,225	1.030 (1.018–1.040)	<0.001	1.020 (1.010–1.031)	<0.001
BUN	4,225	1.290 (1.131–1.477)	<0.001	1.119 (0.960–1.305)	0.150
Creatinine	4,225	1.180 (1.158–1.212)	<0.001	1.018 (0.991–1.046)	0.184

Variables included in the Cox proportional hazards model were the VIS (in the second hour after onset), the NEE score (in the second hour after onset), gender, age, SOFA at onset, APACHE II at admission, the CCI, and other variables at onset, including heart rate, MAP, respiratory rate, SpO_2_, WBC, hematocrit, hemoglobin, platelets, BUN, and creatinine.

### 3.4 Stratified analysis

**Figure 5:**
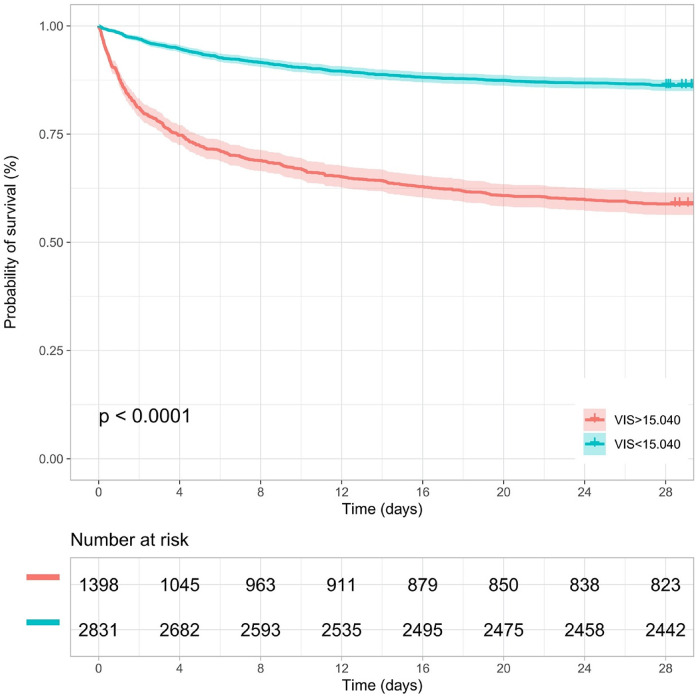
Kaplan–Meier survival curve of the VIS group in the second hour.

**Figure 6:**
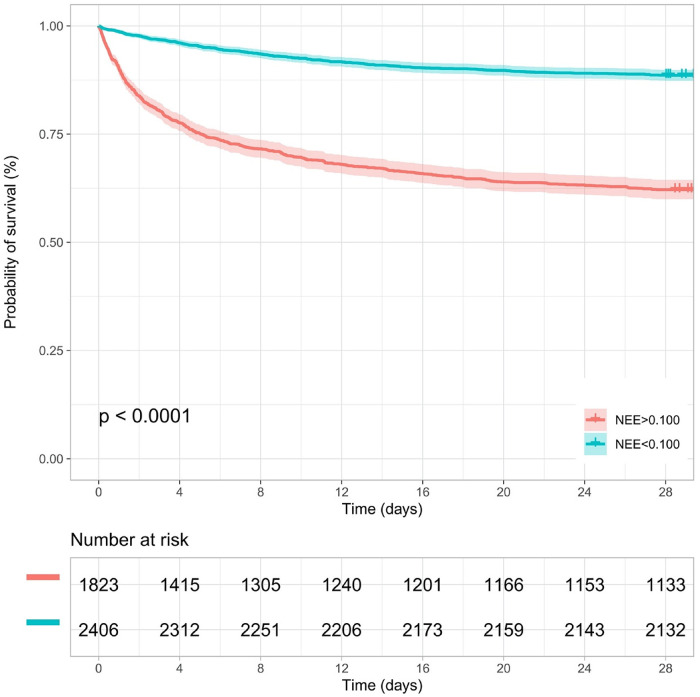
Kaplan–Meier survival curve of the NEE score group in the second hour.

**Figure 7:**
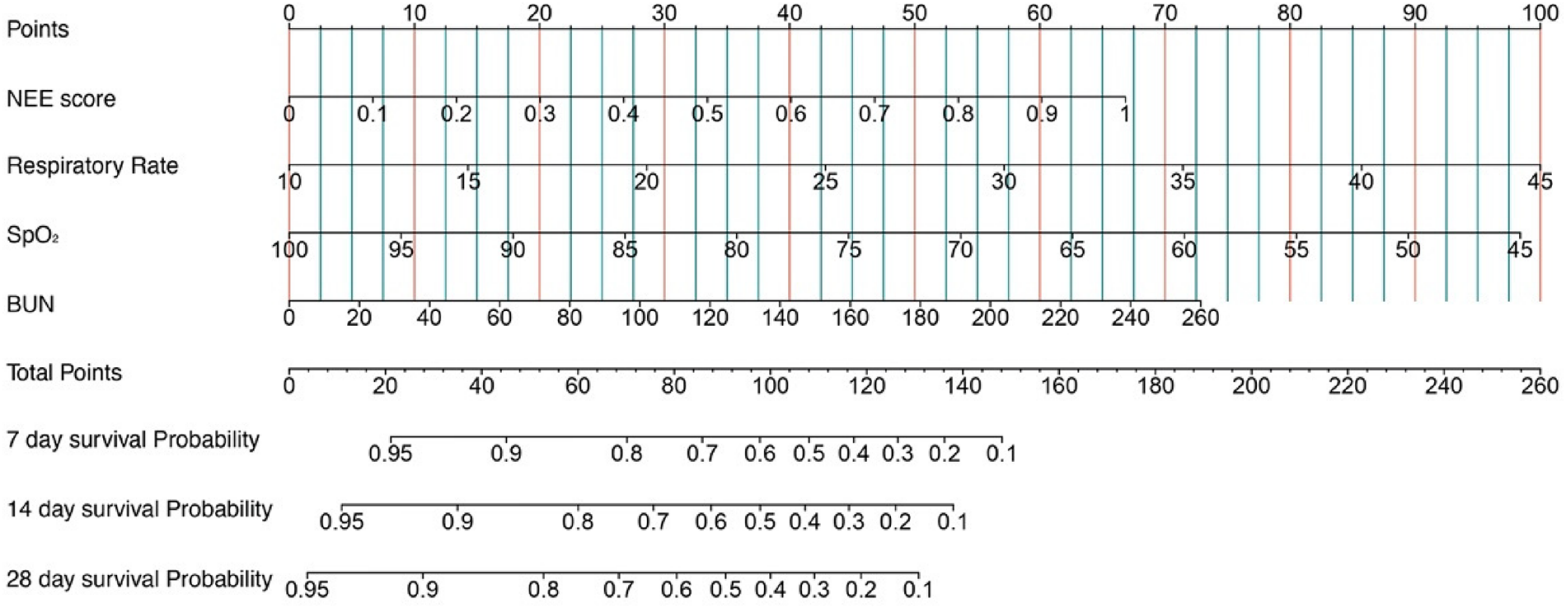
A constructed nomogram for the prognostic prediction of a patient with sepsis based on the NEE score in the second hour.

**Figure 8:**
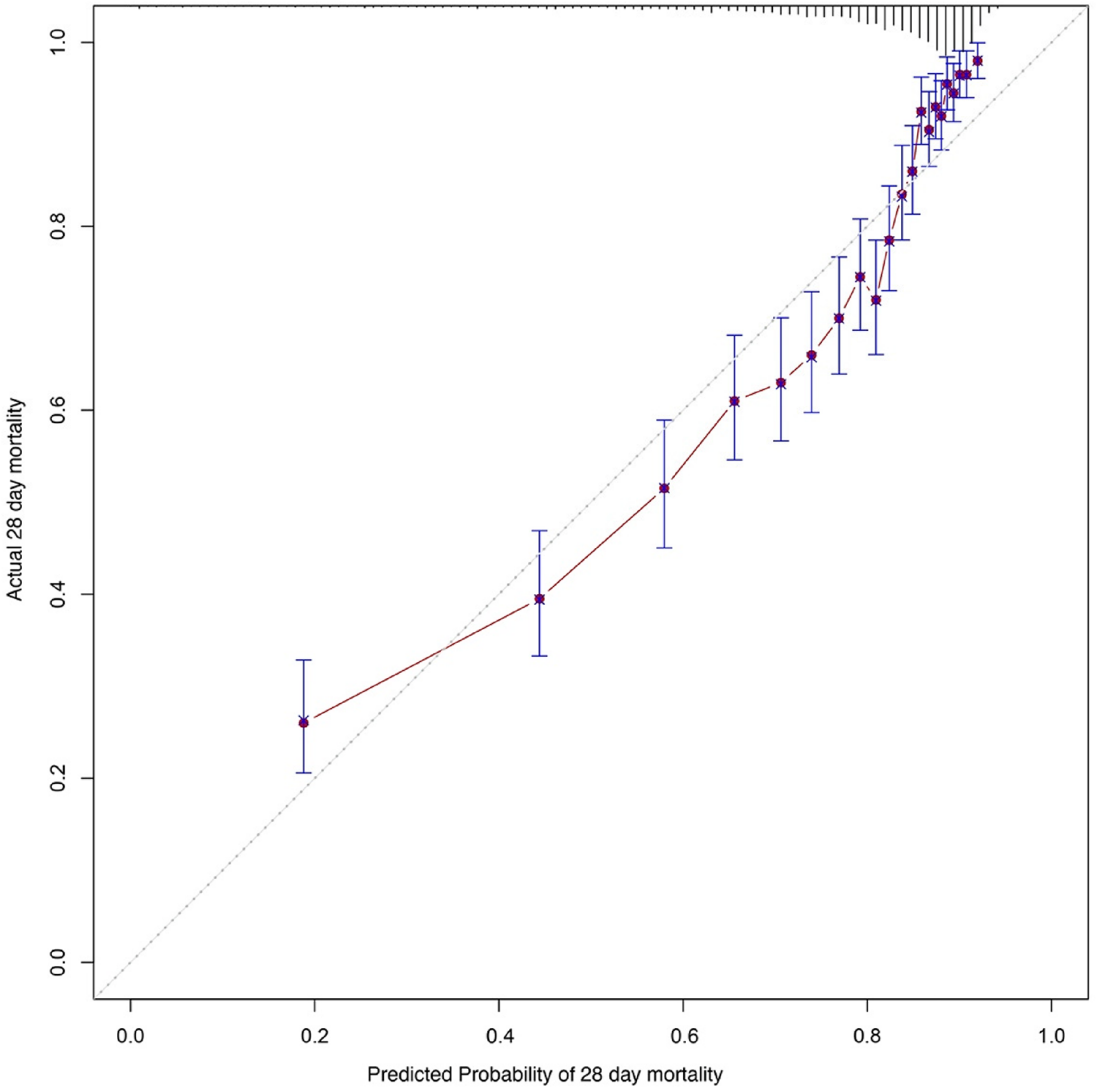
Calibration curves of the prognostic prediction of the nomogram based on the NEE score in the second hour.

**Figure 9:**
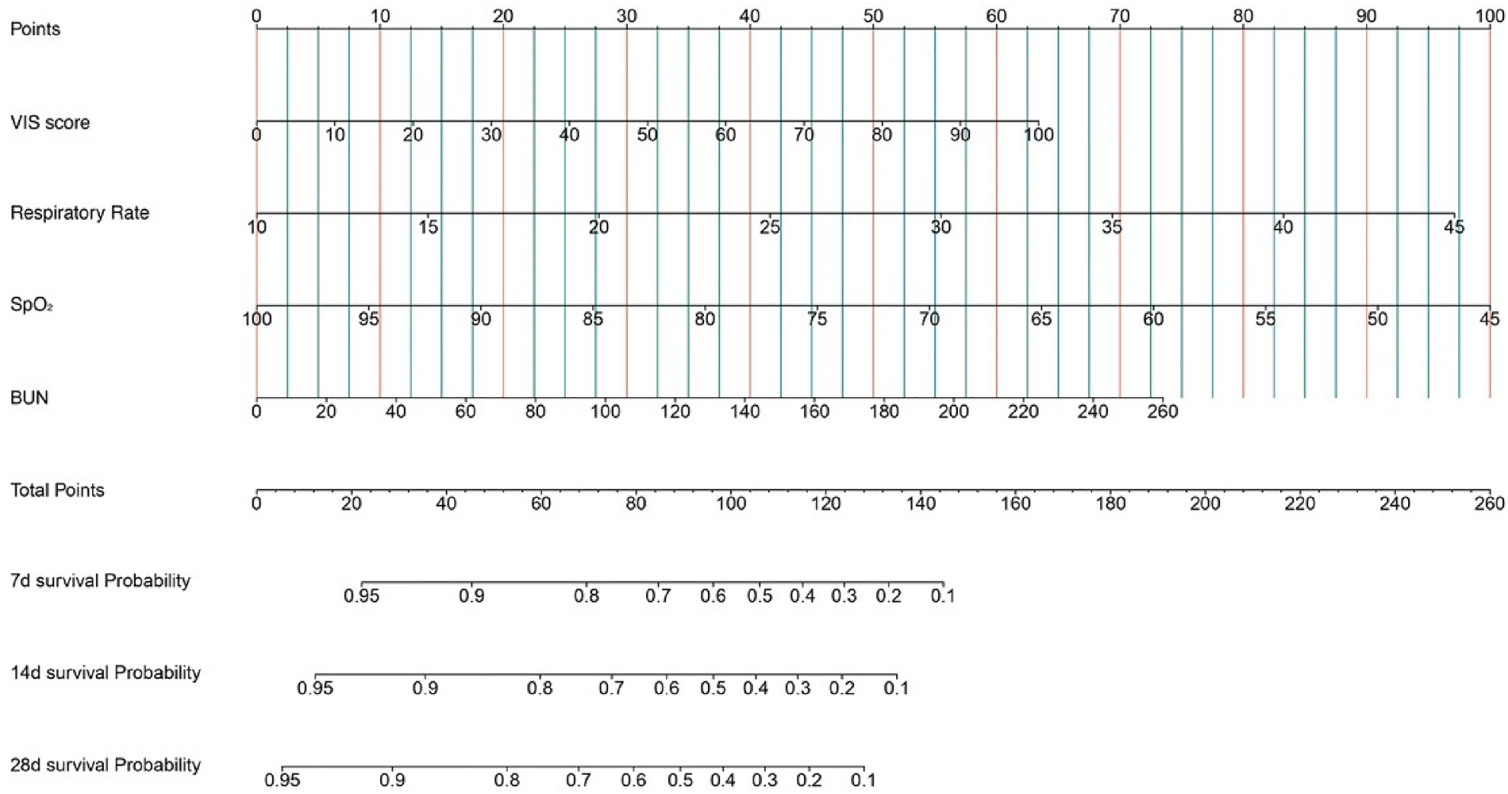
A constructed nomogram for the prognostic prediction of a patient with sepsis based on the VIS in the second hour.

Kaplan–Meier survival analyses were conducted to assess the predictive value of the VIS and NEE score for primary outcomes across the study patients (Figures 5, 6). A significant difference was found in the 28-day mortality between VIS >15.04 and <15.04 (*p* < 0.0001) and between NEE score >0.10 and <0.10 (*p* < 0.0001). The patients in the high VIS and NEE score groups had an increased risk of 28-day mortality. According to the contribution of each influencing factor to the outcome, the nomogram assigned points to each value level of each influencing factor and calculated the predicted value of the individual outcome using the backward method. NEE score, respiratory rate, oxygen saturation, and BUN were eventually incorporated into the nomogram model. The total score of the model ranged from 4 to 148 points and the corresponding risk ratio was 0.05–0.90, and the higher the score value, the higher the risk of death in patients with sepsis (Figure 7). The bootstrap method was used to verify the performance of the prediction model, and the calibration plot showed that the calibration curve fitted well with the ideal curve, with a concordance index of 0.779 (Figure 8). The AUC was 0.802 (95% CI 0.787–0.818), indicating an ideal predictive value of the model (Supplementary Figure S6). The VIS-based nomogram model also had a good predictive value for the prognosis of patients with sepsis (Figure 9; Supplementary Figures S7, S8).

## 4 Discussion

In this retrospective clinical trial involving adult patients with sepsis who received vasoactive therapy, we found significant differences in vasoactive medication exposure between the survival and non-survival groups. In addition, high doses of vasoactive agent exposure quantified by the VIS and NEE score at the second hour after the onset of sepsis were significantly associated with primary and secondary outcomes. At the second hour after sepsis onset, VIS >15.04 and NEE score >0.10 had favorable values for predicting 28-day all-cause mortality, with an AUC of 0.722 and 0.749, respectively.

Since the “inotropic score” (IS) was first proposed in 1995 with the aim of objectively quantifying the level of vasoactive and inotropic drug use in various clinical situations and studies, an increasing number of vasoactive agents have been gradually added to the formula based on relevant research evidence and have evolved into the VIS used currently (13, 16). Similarly, the NEE score is a new scale for quantitatively assessing vasoactive agents that converts the dose of each vasopressor to a dose equivalent to that of norepinephrine (14). However, to date, there is little evidence of the application of this scale in patients with sepsis. Our results revealed that the VIS and NEE score are suitable for mortality prediction and the quantification of various vasopressors, consistent with their application in septic shock patients in the pediatric ICU and emergency department (17, 18).

The VIS has also been used in pediatric and postoperative cardiac surgery patients. A peak VIS during the first 24 h was associated with higher mortality in patients who received out-of-hospital cardiopulmonary resuscitation (19). The VIS has been validated as a predictor of acute kidney injury (AKI) in perioperative patients after cardiovascular surgery. Patients with a higher VIS after cardiac surgery have a higher incidence of AKI (20). The VIS is independently associated with mortality in pediatric septic shock patients (17, 21). The cutoff value of the VIS here was lower than the prediction threshold in pediatric patients with sepsis (15.04 vs. 42.5) (17). This may be due to the large heterogeneity between pediatric and adult patients (13). A NEE of at least 5 μg of norepinephrine or the equivalent per minute was commonly used as an inclusion criterion for septic shock patients in previous studies, almost the same as the cutoff threshold in our results (22). In addition, the NEE score is a vital baseline variable to consider in critical care medicine studies, constantly evolving with changing conversion criteria for vasoactive drug dosages (14, 23). Here, the VIS and NEE score were calculated using continuous infusion doses at each point in time, taking into consideration the changes in some vasoactive drugs along with dose titration. Our results facilitate the use of the VIS and NEE score in this clinical scenario.

Although significant advancements have been achieved in the management of sepsis, several controversial issues remain. Fluid therapy, which can ameliorate hypotension and improve circulatory blood flow, is the primary treatment for patients with sepsis. Besides fluid resuscitation, sepsis-related systemic vasodilation is another pathophysiological mechanism of sepsis. Early vasopressor use can restore vascular tone and maintain organ perfusion pressure (24). According to sepsis guidelines, numerous vasoactive agents are available at the bedside (7). However, the timing of vasoactive drug administration recommended by these guidelines is inconsistent across periods. The early use of vasoactive therapy can convert non-stress volume to stress volume and increase cardiac output. Therefore, the use of vasoactive agents reduces the occurrence of fluid overload in patients with sepsis (25). However, vasoactive agents may cause severe vasoconstriction, impair microcirculation, and lead to organ dysfunction despite the maintenance of blood pressure (26–28). In addition, the use of vasoactive agents in high doses may increase the incidence of adverse events such as arrhythmias and digital ischemia (29). Patient heterogeneity may lead to different levels of responsiveness to treatment, the VIS and NEE score can help identify unnecessary vasoactive therapy and issue an early warning for high-risk patients.

Owing to the uncertain potential effectiveness and inconsistent combinations of different agents acting on various vascular receptors, the efficacy of vasoactive therapy remains unelucidated, with unpredictable results. Schupp et al. recently investigated the prognostic value of norepinephrine dose for 30-day all-cause mortality in patients with sepsis and septic shock (30). Moreover, the inability of the hemodynamic system to match oxygen delivery and oxygen consumption is the main cause of multiorgan dysfunction and death occurrence in patients with sepsis (31). Quantifying the combinations of various vasopressors and inotropes can help optimize the choice and dose titration of drugs. The use of the VIS and NEE score may also facilitate the escalation and de-escalation of hemodynamic monitoring.

The nomogram objectively quantified the predictive value of the variables included in the Cox regression model for the 28-day mortality risk of these patients, and the model stability and ROC curve analysis also suggested that there was a higher risk of death with increasing disease severity and a higher vasoactive treatment dose received by patients with sepsis. The combination respiratory rate, oxygen saturation, and BUN with the NEE score can reveal the functional status of the lungs and kidneys, and the nomogram may help clinicians assess the disease status of these patients, evaluate the benefit–risk ratio of vasoactive treatment, and formulate individualized treatment plans to improve patient prognosis.

This study has several limitations. First, patients who received high doses of vasoactive therapy may have been more severely affected, and data on relevant hemodynamic changes during the administration of vasoactive medication were not included in the analysis. Furthermore, comorbidities, vital signs, and laboratory test results differed significantly between the two groups, suggesting a more severe condition in the non-survivors. Second, patients may have been treated with vasoactive drugs before the diagnosis of sepsis, and the overall exposure may have impacted mortality. Third, phenylephrine use was much more common than usual in the included patients. This may have been due to an inadequate supply of norepinephrine. Fourth, we did not investigate the effects of vasopressors or inotropes on patient outcomes. Fifth, not all drugs in the NEE score and VIS formulas were used in the study, and those that were not included may have contributed to bias in the results. Finally, owing to confounding factors, the extrapolation of retrospective observational trial findings was limited.

## 5 Conclusion

In summary, exposure to high-dose vasoactive medications is closely related to increased mortality in patients with sepsis who are critically ill. This study revealed that the VIS and NEE score have favorable values for predicting mortality risk in patients with sepsis in the ICU. The VIS and NEE score in the second hour after sepsis onset were independently associated with 28-day mortality in patients with sepsis.

## Data Availability

The datasets presented in this study can be found in online repositories. The names of the repository/repositories and accession number(s) can be found below: the data were available from the MIMIC-IV website at https://mimic.physionet.org/.

## Supplementary material

Supplementary Figure S1 Comparison of the ability to predict 7-day mortality between the VIS and NEE score in the second hour. In the second hour, the AUC, sensitivity, and specificity values for the VIS = 17.416 cutoff value were 75.2%, 75.0%, and 63.4%, respectively. The AUC, sensitivity, and specificity values for the NEE score = 0.120 cutoff value were 77.3%, 70.3%, and 72.2%, respectively. VIS, vasoactive-inotropic score; NEE, norepinephrine equivalent; AUC, area under the receiver operating characteristic curve.

Supplementary Figure S2 Comparison of the ability to predict 14-day mortality between the VIS and NEE score in the second hour. In the second hour, the AUC, sensitivity, and specificity values for the VIS = 15.040 cutoff value were 72.7%, 73.7%, and 61.1%, respectively. The AUC, sensitivity, and specificity values for the NEE score = 0.100 cutoff value were 75.3%, 63.1%, and 75.4%, respectively. VIS, vasoactive-inotropic score; NEE, norepinephrine equivalent; AUC, area under the receiver operating characteristic curve.

Supplementary Figure S3 Comparison of the ability to predict 7-day mortality between the VIS and NEE score in the fourth hour. In the fourth hour, the AUC, sensitivity, and specificity values for the VIS = 15.097 cutoff value were 74.9%, 72.4%, and 67.9%, respectively. The AUC, sensitivity, and specificity values for the NEE score = 0.130 cutoff value were 77.0%, 71.8%, and 70.8%, respectively. VIS, vasoactive-inotropic score; NEE, norepinephrine equivalent; AUC, area under the receiver operating characteristic curve.

Supplementary Figure S4 Comparison of the ability to predict 14-day mortality between the VIS and NEE score in the fourth hour. In the fourth hour, the AUC, sensitivity, and specificity values for the VIS = 15.097 cutoff value were 72.8%, 73.5%, and 63.2%, respectively. The AUC, sensitivity, and specificity values for the NEE score = 0.099 cutoff value were 75.3%, 63.6%, and 76.2%, respectively. VIS, vasoactive-inotropic score; NEE, norepinephrine equivalent; AUC, area under the receiver operating characteristic curve.

Supplementary Figure S5 Forest plots of univariable hazard ratios for the primary endpoint in different variables. HR, hazard ratio; VIS, vasoactive-inotropic score; NEE, norepinephrine equivalent; SOFA, sequential organ failure assessment; APACHE II, Acute Physiology and Chronic Health Evaluation II; CCI, Charlson comorbidity index; MAP, mean arterial pressure; SpO_2_, oxygen saturation; WBC, white blood cell; BUN, blood urea nitrogen.

Supplementary Figure S6 Time-dependent AUC of using the nomogram based on the NEE score to predict overall mortality within 28 days. The red line represents the AUC = 0.802, which is considered ideal. AUC, area under the receiver operating characteristic curve.

Supplementary Figure S7 Calibration curves of the prognostic prediction of the nomogram based on the VIS in the second hour. The light gray line indicates the ideal reference line and the red line represents the performance of the nomogram. The closer the solid red line is to the light gray line, the better the predictive value of the model.

Supplementary Figure S8 Time-dependent AUC of using the nomogram based on the VIS to predict overall mortality within 28 days. The red line represents the AUC = 0.8, which is considered ideal. AUC, area under the receiver operating characteristic curve.

Supplementary Table S1 The predictive value of the VIS and NEE score in each hour for 7-day mortality.

Supplementary Table S2 The predictive value of the VIS and NEE score in each hour for 14-day mortality.

Supplementary Table S3 The predictive value of the VIS and NEE score in each hour for 28-day mortality.
