# Supplementary material for: Prognostic evaluation of the norepinephrine equivalent score and the vasoactive-inotropic score in patients with sepsis and septic shock: a retrospective cohort study
Source: Front Cardiovasc Med. 2024 Aug 2;11:1415769. doi: 10.3389/fcvm.2024.1415769 (PMC11327820; doi:10.3389/fcvm.2024.1415769)
Supplement: Supplementary Table S3 — The predictive value of the VIS and NEE score in each hour for 28-day mortality. [file Datasheet1.docx]

**The Supplementary Material**

**Fig. S1.** Comparison of the ability to predict 7-day mortality between VIS and NEE in the second hour.

**Fig. S2**. Comparison of the ability to predict 14-day mortality between VIS and NEE in the second hour.

**Fig. S3.** Comparison of the ability to predict 7-day mortality between VIS and NEE in the fourth hour.

**Fig. S4.** Comparison of the ability to predict 14-day mortality between VIS and NEE in the fourth hour.

**Fig. S5** Forest plots of univariable hazard ratios for the primary endpoint in different variables.

**Fig. S6** Time-dependent AUC of using the nomogram based on NEE to predict overall mortality within 28 days.

**Fig. S7** Calibration curves of prognostic prediction of nomogram based on VIS in the second hour.

**Fig. S8** Time-dependent AUC of using the nomogram based on VIS to predict overall mortality within 28 days.

**Table S1.** The predictive value of VIS and NEE in each hour for 7-day mortality.

**Table S2.** The predictive value of VIS and NEE in each hour for 14-day mortality.

**Table S3.** The predictive value of VIS and NEE in each hour for 28-day mortality.


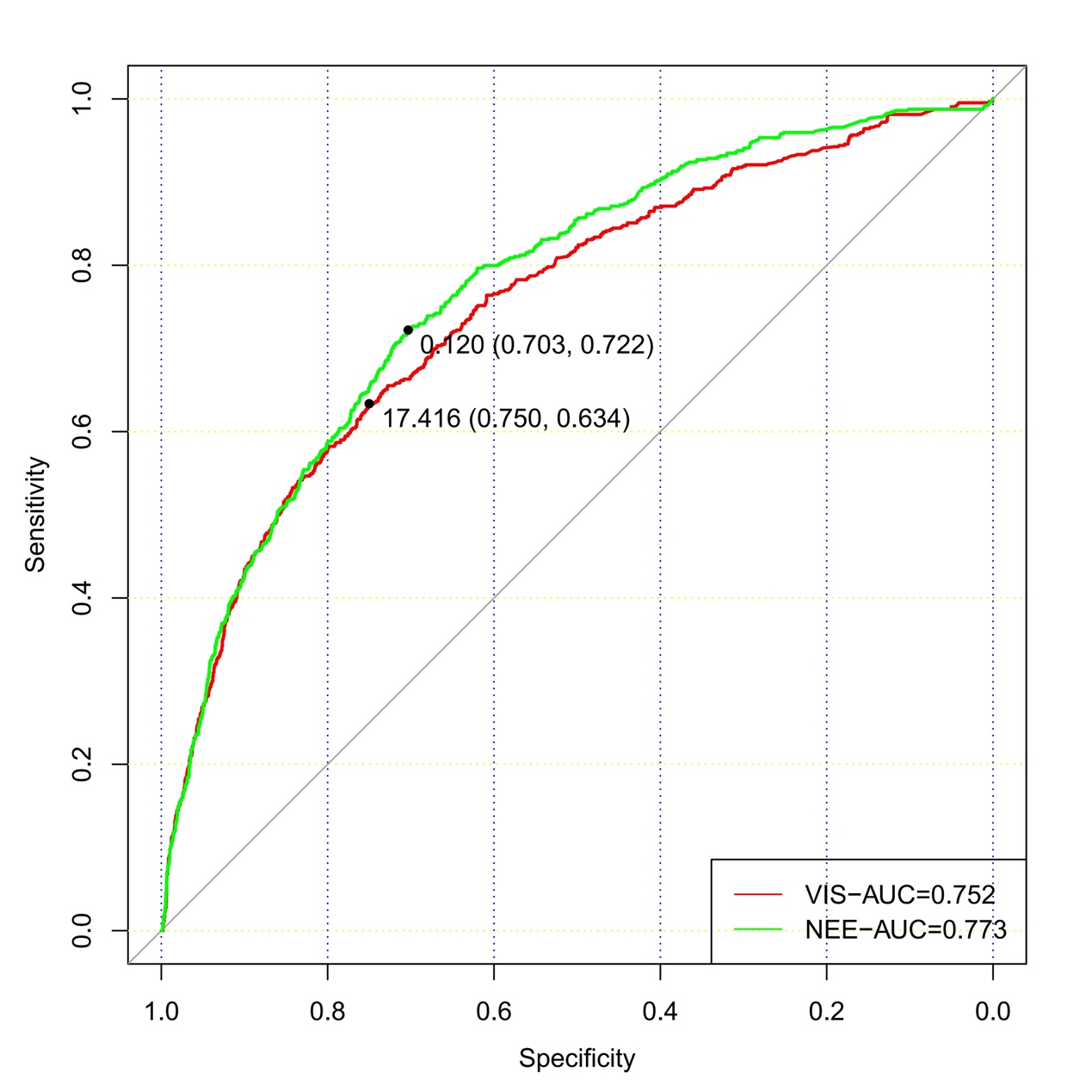


Figure S1. Comparison of the ability to predict 7-day mortality between VIS and NEE in the second hour. In the second hour, The AUC, sensitivity and specificity values for the VIS = 17.416 cut-off value were 75.2%, 75.0% and 63.4%, respectively. The AUC, sensitivity and specificity values for the NEE = 0.120 cut-off value were 77.3%, 70.3% and 72.2%. VIS, vasoactive-inotropic score; NEE, norepinephrine equivalent score; AUC, area under the receiver operating characteristic curve.


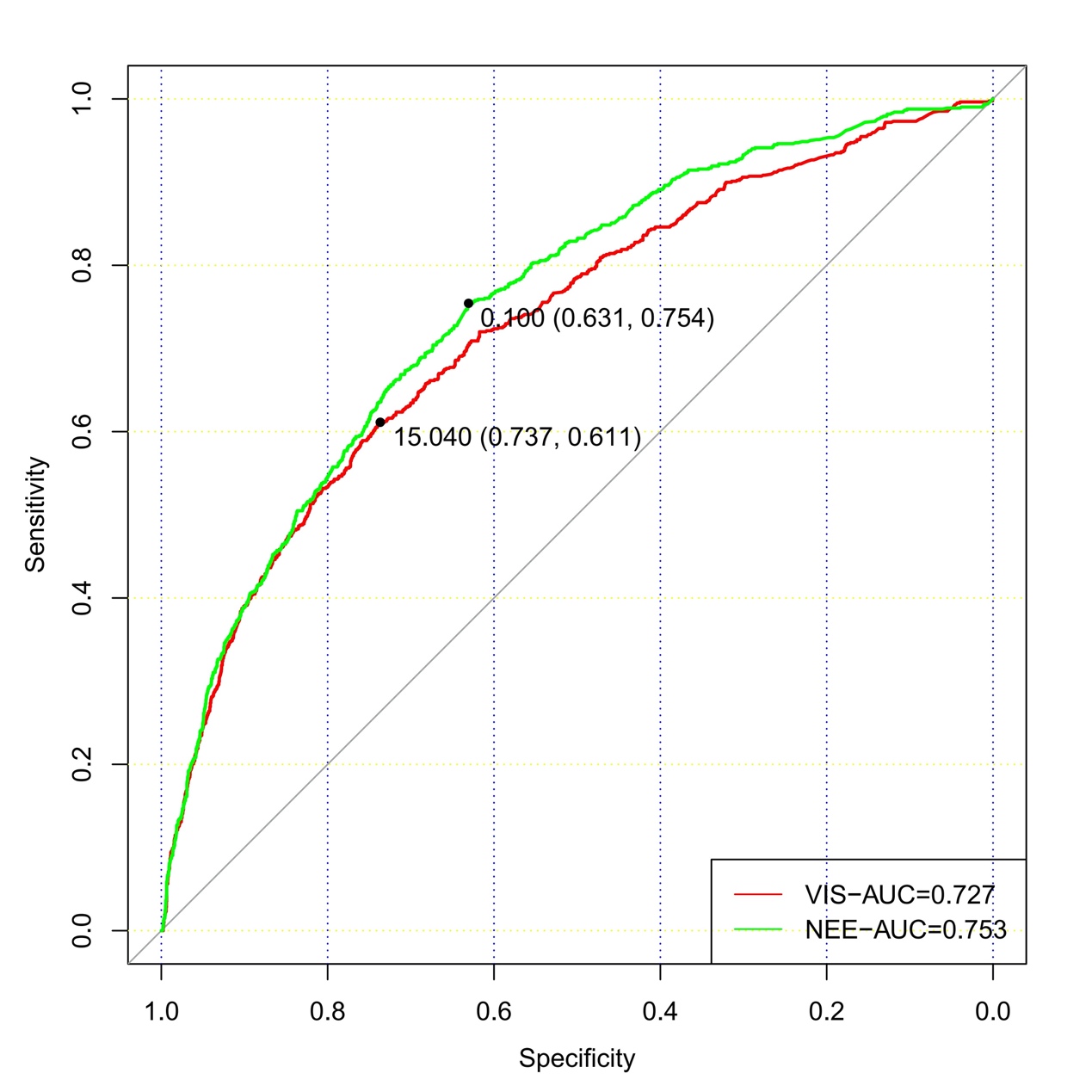


Figure S2. Comparison of the ability to predict 14-day mortality between VIS and NEE in the second hour. In the second hour, The AUC, sensitivity and specificity values for the VIS = 15.040 cut-off value were 72.7%, 73.7% and 61.1%, respectively. The AUC, sensitivity and specificity values for the NEE = 0.100 cut-off value were 75.3%, 63.1% and 75.4%. VIS, vasoactive-inotropic score; NEE, norepinephrine equivalent score; AUC, area under the receiver operating characteristic curve.


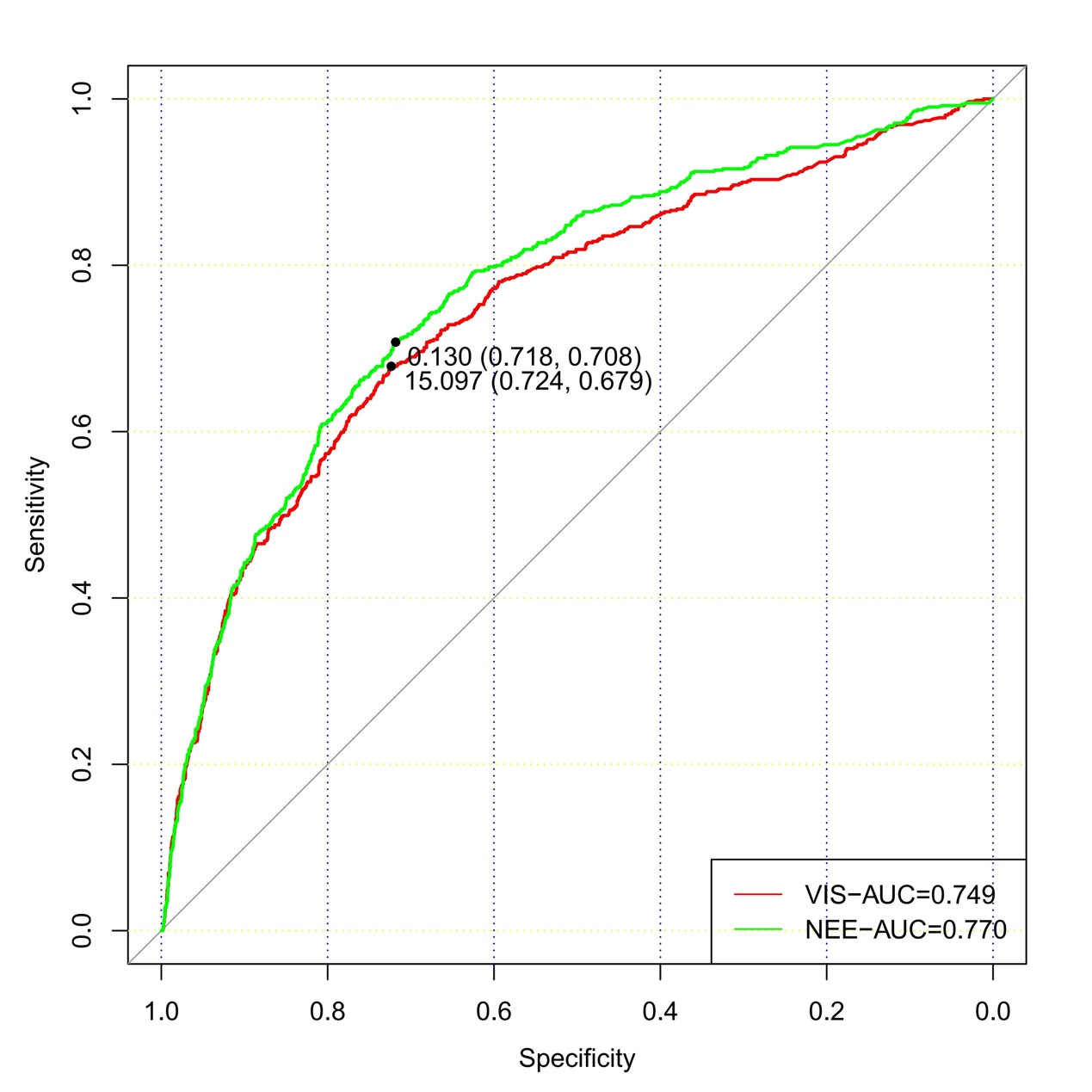


Figure S3. Comparison of the ability to predict 7-day mortality between VIS and NEE in the fourth hour. In the fourth hour, The AUC, sensitivity and specificity values for the VIS = 15.097 cut-off value were 74.9%, 72.4% and 67.9%, respectively. The AUC, sensitivity and specificity values for the NEE = 0.130 cut-off value were 77.0%, 71.8% and 70.8%. VIS, vasoactive-inotropic score; NEE, norepinephrine equivalent score; AUC, area under the receiver operating characteristic curve.


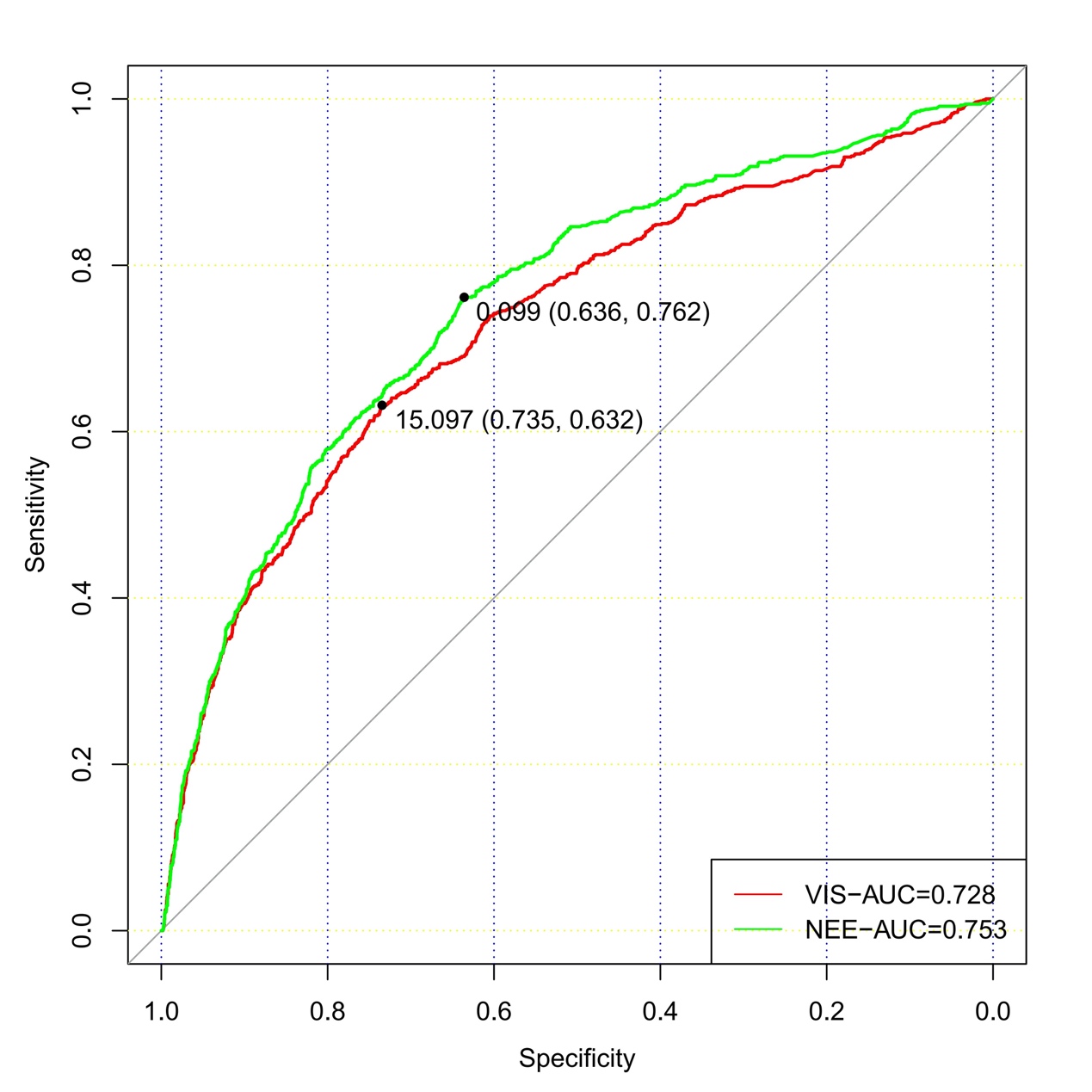


Figure S4. Comparison of the ability to predict 14-day mortality between VIS and NEE in the fourth hour. In the fourth hour, The AUC, sensitivity and specificity values for the VIS = 15.097 cut-off value were 72.8%, 73.5% and 63.2%, respectively. The AUC, sensitivity and specificity values for the NEE = 0.099 cut-off value were 75.3%, 63.6% and 76.2%. VIS, vasoactive-inotropic score; NEE, norepinephrine equivalent score; AUC, area under the receiver operating characteristic curve.


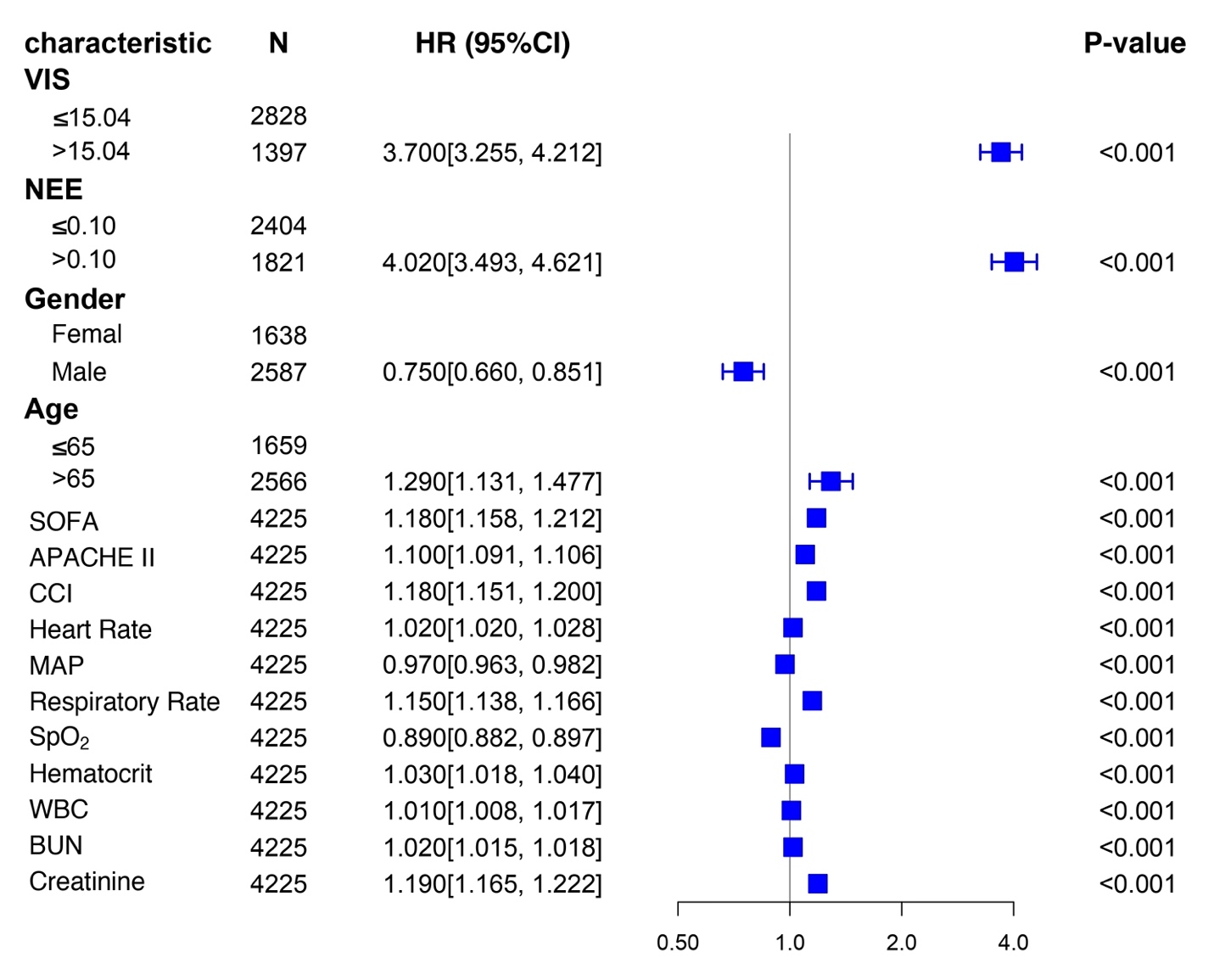


Figure S5. Forest plots of univariable hazard ratios for the primary endpoint in different variables. HR, hazard ratio; VIS, vasoactive-inotropic score; NEE, norepinephrine equivalent score; SOFA, sequential organ failure assessment; APACHE, acute physiology and chronic health evaluation; CCI, charlson comorbidity index; MAP, mean arterial pressure; SpO_2_, oxygen saturation; WBC, white blood cell; BUN, blood urea nitrogen.


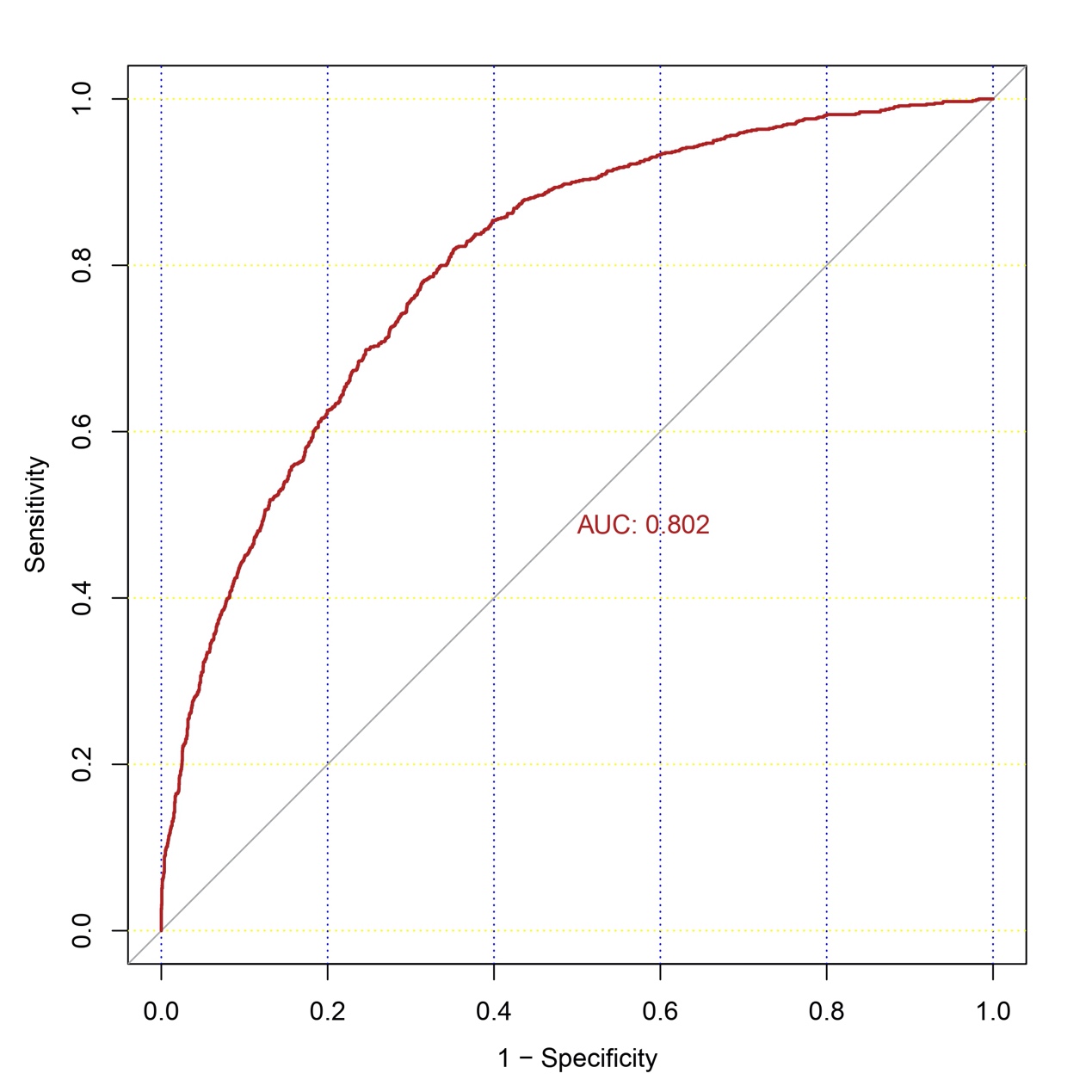


Figure S6. Time-dependent AUC of using the nomogram based on NEE to predict overall mortality within 28 days. The red line represents AUC = 0.802, which is considered ideal. AUC: area under the receiver operating characteristic curves.


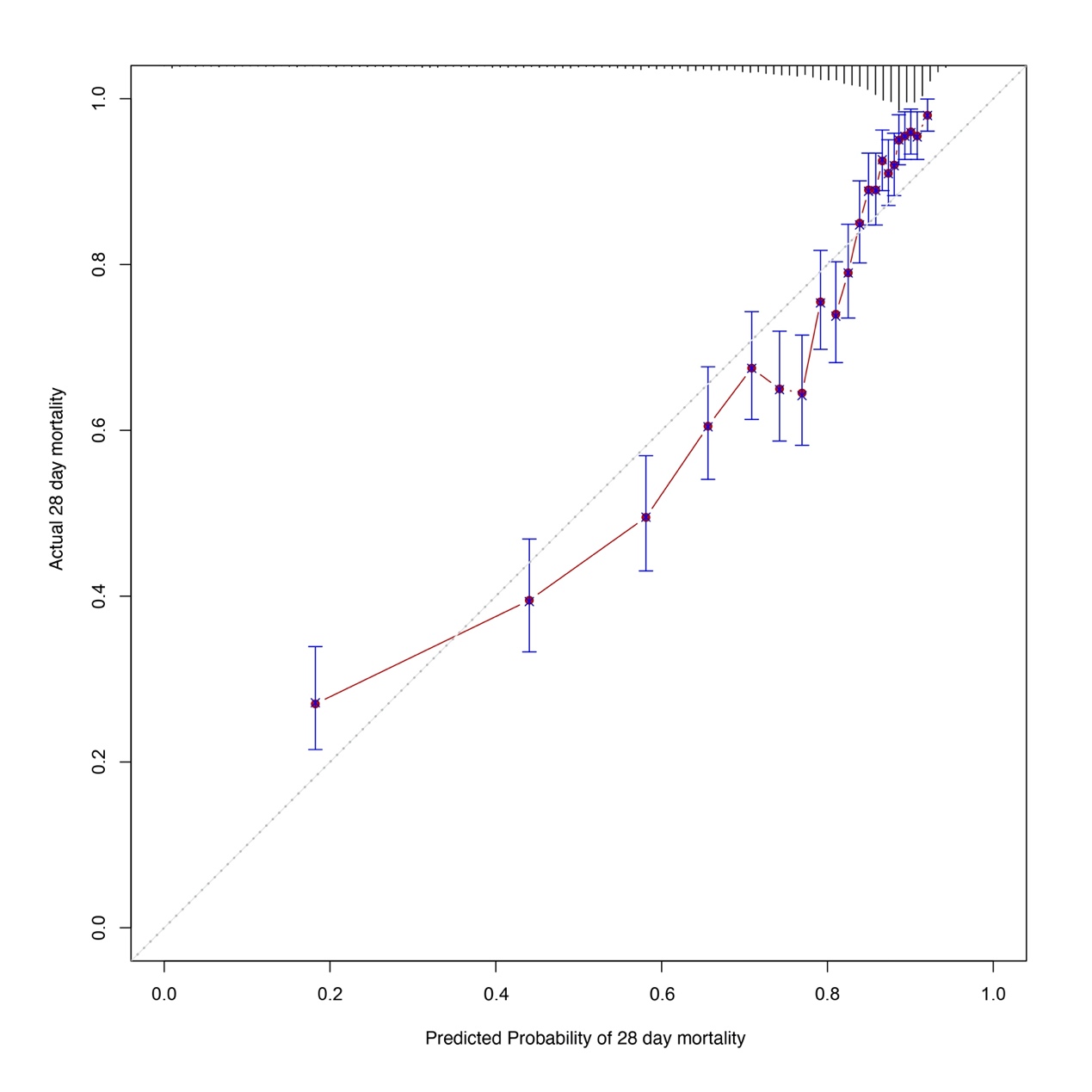


Figure S7. Calibration curves of prognostic prediction of nomogram based on VIS in the second hour. The light grey line indicates the ideal reference line and the red line represents the performance of the nomogram. The closer the solid red line is to the light grey line, the better the predictive value of the model.

**
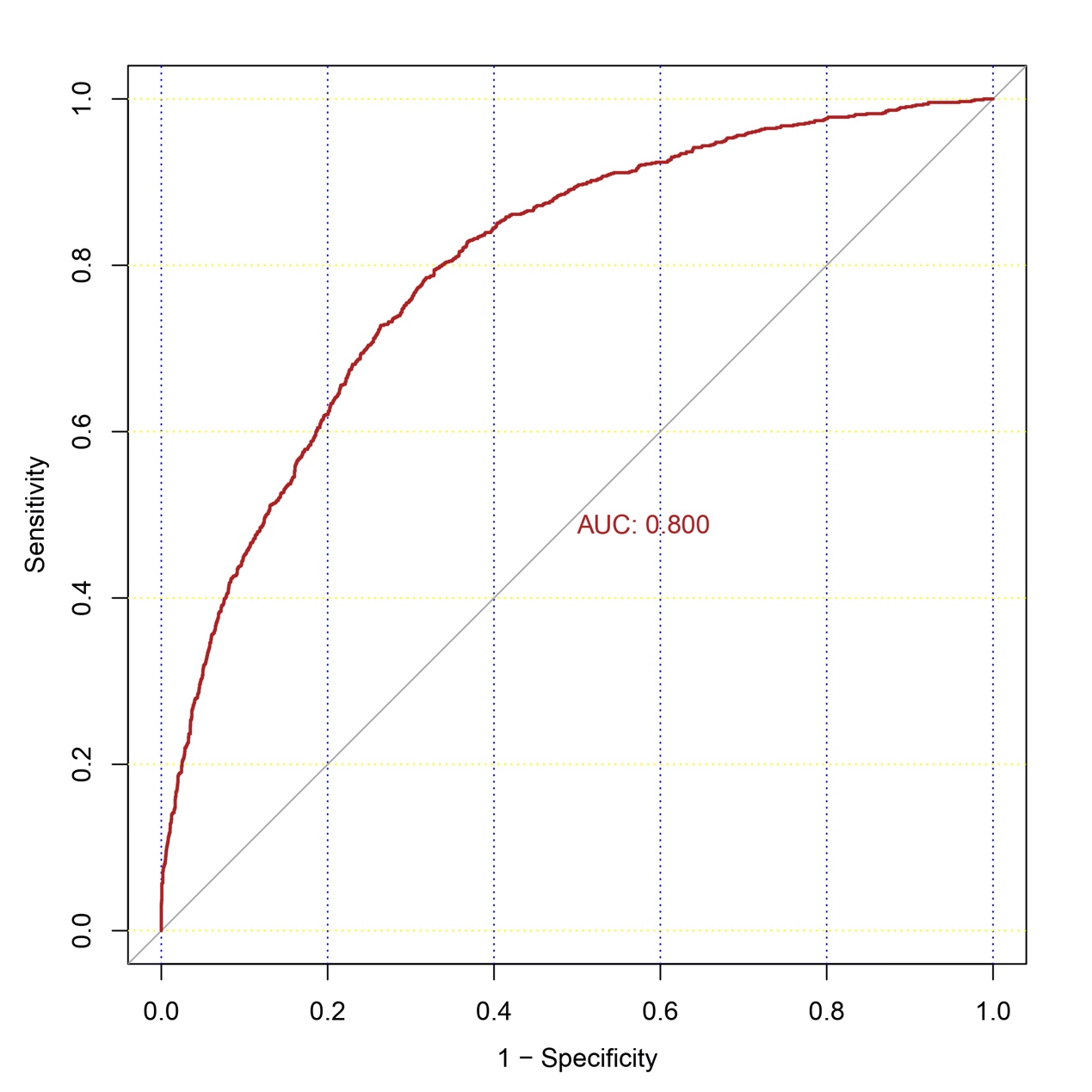
**

Figure S8. Time-dependent AUC of using the nomogram based on VIS to predict overall mortality within 28 days. The red line represents AUC = 0.8, which is considered ideal. AUC: area under the receiver operating characteristic curves.

**Table 1.** Demographic data and baseline characteristics

| Variables | All patients  (n=4229) | Survivors  (n=3265) | Non-survivors  (n=964) | *P*-Value |
| --- | --- | --- | --- | --- |
| Age (yr), mean (SD) | 67.7 (14.4) | 66.9 (14.1) | 70.3 (15.2) | <0.001 |
| Gender (Male), n (%) | 1639 (38.8%) | 1205 (36.9%) | 434 (45.0%) | <0.001 |
| Height (cm), mean (SD) | 170 (10.2) | 170 (10.0) | 168 (10.4) | <0.001 |
| Charlson Comorbidity Index, mean (SD) | 4.99 (2.74) | 4.64 (2.58) | 6.18 (2.94) | <0.001 |
| SOFA, mean (SD) | 4.37 (2.26) | 4.11 (2.02) | 5.25 (2.76) | <0.001 |
| APACHE II, mean (SD) | 21.5 (7.69) | 19.9 (6.82) | 26.9 (7.99) | <0.001 |
| **Comorbidities, n (%)** |  |  |  |  |
| Liver disease | 614 (14.5%) | 354 (10.8%) | 260 (26.9%) | <0.001 |
| Hypertension | 2778 (65.7%) | 2192 (67.2%) | 586 (60.7%) | <0.001 |
| Cerebrovascular disease | 453 (10.7%) | 310 (9.5%) | 143 (14.8%) | <0.001 |
| Chronic pulmonary disease | 1081 (25.6%) | 808 (24.8%) | 273 (28.3%) | 0.03 |
| Congestive heart failure | 1274 (30.1%) | 922 (28.2%) | 352 (36.5%) | <0.001 |
| Peripheral vascular disease | 599 (14.2%) | 458 (14.0%) | 141 (14.6%) | 0.688 |
| Renal disease | 831 (19.7%) | 580 (17.8%) | 251 (26.0%) | <0.001 |
| Metastatic solid tumor | 202 (4.8%) | 105 (3.2%) | 97 (10.1%) | <0.001 |
| Hematologic malignancy | 485 (11.5%) | 317 (9.7%) | 168 (17.4%) | <0.001 |
| **Vital signs, mean (SD)** |  |  |  |  |
| Heart rate (beats/min) | 71.1 (15.1) | 70.4 (13.8) | 73.4 (18.6) | <0.001 |
| Mean arterial pressure (mmHg) | 73.4 (7.41) | 73.7 (6.67) | 72.3 (9.42) | <0.001 |
| Respiratory rate (breaths/min) | 19.7 (4.17) | 18.9 (3.73) | 22.1 (4.62) | <0.001 |
| Body temperature (­°C) | 36.8 (0.70) | 36.9 (0.59) | 36.6 (0.97) | <0.001 |
| Oxygen saturation (%) | 97.1 (3.13) | 97.5 (1.78) | 95.5 (5.38) | <0.001 |
| **Laboratory tests, mean (SD)** |  |  |  |  |
| WBC (10^9^/L) | 17.8 (10.9) | 17.4 (9.24) | 19.3 (15.1) | <0.001 |
| Hematocrit (%) | 28.6 (6.02) | 28.3 (5.61) | 29.4 (7.17) | <0.001 |
| Hemoglobin (g/dL) | 9.50 (1.97) | 9.49 (1.84) | 9.54 (2.35) | 0.538 |
| Platelet (10^9^/L) | 160 (89.7) | 160 (84.0) | 160 (107) | 0.866 |
| BUN (mg/dL) | 31.1 (24.2) | 27.2 (20.8) | 44.4 (29.4) | <0.001 |
| Creatinine (mg/dL) | 1.75 (1.59) | 1.56 (1.49) | 2.39 (1.73) | <0.001 |
| **Vasopressors use in the second hour,** **median (IQR)** |  |  |  |  |
| Norepinephrine dose (µg/kg/min) | 0.140 [0.209] | 0.100 [0.140] | 0.200 [0.281] | 0.204 |
| Epinephrine dose (µg/kg/ min) | 0.040 [0.060] | 0.030 [0.030] | 0.103 [0.151] | <0.001 |
| Dopamine dose (µg/kg/min) | 8.04 [10.00] | 7.51 [5.22] | 10.0 [14.20] | 0.021 |
| Phenylephrine dose (µg/kg/min) | 0.800 [1.00] | 0.700 [0.502] | 2.00 [3.000] | <0.001 |
| Vasopressin dose (U/min) | 2.40 [0.007] | 2.40 [0.014] | 2.40 [0.005] | 0.213 |
| Angiotensin II dose (ng/kg/min) | 0.020 [0.001] |  | 0.020 [0.001] |  |
| Dobutamine (µg/kg/min) | 5.00 [4.26] | 5.00 [2.51] | 5.01 [5.38] | 0.094 |
| Milrinone (µg/kg/min) | 0.800 [1.00] | 0.700 [0.502] | 2.00 [3.00] | 0.295 |
| **VIS and NEE in the second hour, median (IQR)** |  |  |  |  |
| VIS | 10.0 [15.0] | 9.01 [10.3] | 20.0 [32.0] | <0.001 |
| NEE | 0.080 [0.160] | 0.060 [0.107] | 0.200 [0.311] | <0.001 |

APACHE, acute physiology and chronic health evaluation; SD, standard deviation; SOFA, Sequential Organ Failure Assessment; WBC, white blood cell; BUN, blood urea nitrogen; VIS, vasoactive-inotropic score; NEE, norepinephrine equivalent score.

**Table 2.** Predictors of 28-day mortality using the Cox proportional hazard model.

| **Characteristic** | **N** | **Univariable HR (95%CI)** | **P-value** | **Multivariable HR (95%CI)** | **P-value** |
| --- | --- | --- | --- | --- | --- |
| VIS |  |  |  |  |  |
| ≤15.04 | 2828 | 1 (Reference group) |  | 1 (Reference group) |  |
| >15.04 | 1397 | 3.700[3.255, 4.212] | <0.001 | 1.402[1.147, 1.714] | 0.001 |
| NEE |  |  |  |  |  |
| ≤0.10 | 2404 | 1 (Reference group) |  | 1 (Reference group) |  |
| >0.10 | 1821 | 4.020[3.493, 4.621] | <0.001 | 1.600[1.284, 1.994] | <0.001 |
| Gender |  |  |  |  |  |
| Female | 1638 | 1 (Reference group) |  | 1 (Reference group) |  |
| Male | 2587 | 0.750[0.660, 0.851] | <0.001 | 0.767[0.674, 0.874] | <0.001 |
| Age |  |  |  |  |  |
| ≤65 | 1659 | 1 (Reference group) |  | 1 (Reference group) |  |
| >65 | 2566 | 1.290[1.131, 1.477] | <0.001 | 1.119[0.960, 1.305] | 0.150 |
| SOFA | 4225 | 1.180[1.158, 1.212] | <0.001 | 1.018[0.991, 1.046] | 0.184 |
| APACHE II | 4225 | 1.100[1.091, 1.106] | <0.001 | 1.048[1.038, 1.058] | <0.001 |
| CCI | 4225 | 1.180[1.151, 1.200] | <0.001 | 1.116[1.089, 1.144] | <0.001 |
| Heart Rate | 4225 | 1.020[1.020, 1.028] | <0.001 | 1.004[1.000, 1.008] | 0.044 |
| MAP | 4225 | 0.970[0.963, 0.982] | <0.001 | 0.988[0.979, 0.997] | 0.009 |
| Respiratory Rate | 4225 | 1.150[1.138, 1.166] | <0.001 | 1.062[1.044, 1.080] | <0.001 |
| SpO_2_ | 4225 | 0.890[0.882, 0.897] | <0.001 | 0.939[0.927, 0.950] | <0.001 |
| WBC | 4225 | 1.010[1.008, 1.017] | <0.001 | 0.998[0.992, 1.003] | 0.440 |
| Hematocrit | 4225 | 1.030[1.018, 1.040] | <0.001 | 1.020[1.010, 1.031] | <0.001 |
| BUN | 4225 | 1.290[1.131, 1.477] | <0.001 | 1.119[0.960, 1.305] | 0.150 |
| Creatinine | 4225 | 1.180[1.158, 1.212] | <0.001 | 1.018[0.991, 1.046] | 0.184 |

Variables included in Cox proportional hazards model were VIS (in the second hour after onset), NEE (in the second hour after onset), gender, age, SOFA at onset, APACHE II at admission, CCI, and other variables at onset including heart rate, MAP, respiratory rate, SpO_2_, WBC, hematocrit, hemoglobin, platelets, BUN, creatinine. HR, hazard ratio; VIS, vasoactive-inotropic score; NEE, norepinephrine equivalent score; SOFA, sequential organ failure assessment; APACHE, acute physiology and chronic health evaluation; CCI, charlson comorbidity index; MAP, mean arterial pressure; SpO_2_, oxygen saturation; WBC, white blood cell; BUN, blood urea nitrogen.

**Table S1.** The predictive value of VIS and NEE in each hour for 7-day mortality.

| **Time point** | **VIS** | | | | **NEE** | | | |
| --- | --- | --- | --- | --- | --- | --- | --- | --- |
|  | **AUC** | **Cutoff value** | **Sensitivity** | **Specificity** | **AUC** | **Cutoff value** | **Sensitivity** | **Specificity** |
| 1h | 0.741 | 13.024 | 0.675 | 0.707 | 0.763 | 0.120 | 0.717 | 0.700 |
| 2h | 0.752 | 17.416 | 0.750 | 0.634 | 0.773 | 0.120 | 0.703 | 0.722 |
| 3h | 0.739 | 20.034 | 0.809 | 0.560 | 0.762 | 0.120 | 0.697 | 0.699 |
| 4h | 0.749 | 15.097 | 0.724 | 0.679 | 0.770 | 0.130 | 0.718 | 0.708 |
| 5h | 0.740 | 15.018 | 0.707 | 0.671 | 0.763 | 0.120 | 0.694 | 0.720 |
| 6h | 0.736 | 22.842 | 0.813 | 0.561 | 0.761 | 0.120 | 0.678 | 0.716 |
| 7h | 0.738 | 20.031 | 0.784 | 0.592 | 0.738 | 0.150 | 0.735 | 0.668 |
| 8h | 0.719 | 17.744 | 0.713 | 0.630 | 0.741 | 0.120 | 0.646 | 0.715 |
| 9h | 0.721 | 22.069 | 0.801 | 0.547 | 0.742 | 0.120 | 0.659 | 0.702 |
| 10h | 0.722 | 20.102 | 0.785 | 0.561 | 0.735 | 0.150 | 0.704 | 0.644 |
| 11h | 0.713 | 15.656 | 0.691 | 0.649 | 0.725 | 0.150 | 0.689 | 0.648 |
| 12h | 0.701 | 18.116 | 0.726 | 0.591 | 0.719 | 0.180 | 0.752 | 0.589 |
| 13h | 0.708 | 15.082 | 0.686 | 0.651 | 0.724 | 0.180 | 0.749 | 0.591 |
| 14h | 0.705 | 19.991 | 0.749 | 0.580 | 0.719 | 0.130 | 0.662 | 0.670 |
| 15h | 0.689 | 17.460 | 0.722 | 0.575 | 0.706 | 0.179 | 0.760 | 0.555 |
| 16h | 0.711 | 15.135 | 0.678 | 0.660 | 0.728 | 0.160 | 0.723 | 0.639 |
| 17h | 0.708 | 17.414 | 0.709 | 0.607 | 0.724 | 0.159 | 0.709 | 0.622 |
| 18h | 0.728 | 13.473 | 0.644 | 0.706 | 0.743 | 0.100 | 0.561 | 0.810 |
| 19h | 0.716 | 20.113 | 0.793 | 0.562 | 0.731 | 0.190 | 0.784 | 0.586 |
| 20h | 0.714 | 18.029 | 0.729 | 0.608 | 0.730 | 0.092 | 0.545 | 0.802 |
| 21h | 0.680 | 20.004 | 0.766 | 0.526 | 0.690 | 0.150 | 0.686 | 0.603 |
| 22h | 0.675 | 14.636 | 0.653 | 0.619 | 0.693 | 0.096 | 0.535 | 0.759 |
| 23h | 0.690 | 19.970 | 0.762 | 0.555 | 0.702 | 0.113 | 0.602 | 0.733 |
| 24h | 0.689 | 16.939 | 0.710 | 0.603 | 0.702 | 0.120 | 0.609 | 0.700 |

**Table S2.** The predictive value of VIS and NEE in each hour for 14-day mortality.

| **Time point** | **VIS** | | | | **NEE** | | | |
| --- | --- | --- | --- | --- | --- | --- | --- | --- |
|  | **AUC** | **Cutoff value** | **Sensitivity** | **Specificity** | **AUC** | **Cutoff value** | **Sensitivity** | **Specificity** |
| 1h | 0.720 | 13.024 | 0.684 | 0.663 | 0.747 | 0.094 | 0.625 | 0.757 |
| 2h | 0.727 | 15.040 | 0.737 | 0.611 | 0.753 | 0.100 | 0.631 | 0.754 |
| 3h | 0.717 | 15.066 | 0.733 | 0.600 | 0.743 | 0.096 | 0.631 | 0.738 |
| 4h | 0.728 | 15.097 | 0.735 | 0.632 | 0.753 | 0.099 | 0.636 | 0.762 |
| 5h | 0.724 | 15.018 | 0.717 | 0.640 | 0.748 | 0.100 | 0.634 | 0.759 |
| 6h | 0.722 | 18.005 | 0.743 | 0.609 | 0.749 | 0.120 | 0.675 | 0.703 |
| 7h | 0.719 | 20.020 | 0.790 | 0.557 | 0.744 | 0.130 | 0.704 | 0.674 |
| 8h | 0.710 | 17.744 | 0.724 | 0.596 | 0.731 | 0.121 | 0.675 | 0.674 |
| 9h | 0.708 | 16.282 | 0.717 | 0.612 | 0.731 | 0.140 | 0.697 | 0.649 |
| 10h | 0.704 | 15.056 | 0.701 | 0.620 | 0.721 | 0.150 | 0.719 | 0.619 |
| 11h | 0.705 | 15.656 | 0.705 | 0.624 | 0.719 | 0.096 | 0.563 | 0.770 |
| 12h | 0.697 | 16.212 | 0.713 | 0.590 | 0.718 | 0.180 | 0.766 | 0.563 |
| 13h | 0.690 | 15.219 | 0.699 | 0.606 | 0.708 | 0.150 | 0.710 | 0.600 |
| 14h | 0.698 | 15.272 | 0.703 | 0.615 | 0.713 | 0.130 | 0.681 | 0.656 |
| 15h | 0.689 | 17.460 | 0.722 | 0.575 | 0.706 | 0.179 | 0.760 | 0.555 |
| 16h | 0.695 | 20.127 | 0.793 | 0.522 | 0.716 | 0.140 | 0.696 | 0.641 |
| 17h | 0.694 | 17.305 | 0.721 | 0.572 | 0.714 | 0.095 | 0.539 | 0.783 |
| 18h | 0.708 | 18.020 | 0.750 | 0.578 | 0.723 | 0.100 | 0.563 | 0.773 |
| 19h | 0.695 | 20.011 | 0.791 | 0.515 | 0.715 | 0.127 | 0.687 | 0.644 |
| 20h | 0.703 | 16.886 | 0.721 | 0.585 | 0.721 | 0.099 | 0.573 | 0.776 |
| 21h | 0.664 | 14.997 | 0.670 | 0.588 | 0.676 | 0.150 | 0.696 | 0.580 |
| 22h | 0.672 | 12.625 | 0.625 | 0.638 | 0.690 | 0.096 | 0.550 | 0.734 |
| 23h | 0.673 | 19.896 | 0.773 | 0.512 | 0.688 | 0.113 | 0.622 | 0.694 |
| 24h | 0.683 | 15.195 | 0.692 | 0.600 | 0.698 | 0.110 | 0.616 | 0.693 |

**Table S3.** The predictive value of VIS and NEE in each hour for 28-day mortality.

| **Time point** | **VIS** | | | | **NEE** | | | |
| --- | --- | --- | --- | --- | --- | --- | --- | --- |
|  | **AUC** | **Cutoff value** | **Sensitivity** | **Specificity** | **AUC** | **Cutoff value** | **Sensitivity** | **Specificity** |
| 1h | 0.714 | 13.024 | 0.694 | 0.647 | 0.743 | 0.096 | 0.639 | 0.742 |
| 2h | 0.722 | 15.040 | 0.748 | 0.596 | 0.749 | 0.100 | 0.643 | 0.738 |
| 3h | 0.716 | 15.066 | 0.745 | 0.593 | 0.741 | 0.100 | 0.648 | 0.724 |
| 4h | 0.721 | 15.097 | 0.747 | 0.616 | 0.747 | 0.099 | 0.648 | 0.741 |
| 5h | 0.725 | 15.018 | 0.730 | 0.627 | 0.749 | 0.120 | 0.720 | 0.674 |
| 6h | 0.713 | 19.988 | 0.778 | 0.561 | 0.741 | 0.120 | 0.684 | 0.688 |
| 7h | 0.739 | 19.999 | 0.788 | 0.548 | 0.713 | 0.130 | 0.718 | 0.656 |
| 8h | 0.704 | 17.744 | 0.736 | 0.579 | 0.725 | 0.120 | 0.675 | 0.665 |
| 9h | 0.699 | 22.069 | 0.821 | 0.492 | 0.722 | 0.096 | 0.587 | 0.747 |
| 10h | 0.698 | 15.056 | 0.712 | 0.602 | 0.717 | 0.150 | 0.733 | 0.595 |
| 11h | 0.697 | 15.656 | 0.716 | 0.600 | 0.713 | 0.096 | 0.577 | 0.750 |
| 12h | 0.694 | 16.212 | 0.726 | 0.576 | 0.713 | 0.180 | 0.778 | 0.544 |
| 13h | 0.682 | 15.637 | 0.714 | 0.582 | 0.701 | 0.150 | 0.722 | 0.577 |
| 14h | 0.685 | 15.272 | 0.713 | 0.584 | 0.703 | 0.130 | 0.690 | 0.624 |
| 15h | 0.683 | 14.996 | 0.681 | 0.604 | 0.701 | 0.160 | 0.750 | 0.550 |
| 16h | 0.686 | 15.928 | 0.708 | 0.589 | 0.707 | 0.093 | 0.562 | 0.756 |
| 17h | 0.689 | 15.347 | 0.699 | 0.578 | 0.710 | 0.100 | 0.562 | 0.755 |
| 18h | 0.700 | 18.020 | 0.759 | 0.549 | 0.714 | 0.099 | 0.575 | 0.752 |
| 19h | 0.683 | 20.005 | 0.800 | 0.490 | 0.703 | 0.127 | 0.700 | 0.620 |
| 20h | 0.698 | 15.275 | 0.711 | 0.591 | 0.716 | 0.090 | 0.574 | 0.769 |
| 21h | 0.653 | 14.996 | 0.681 | 0.570 | 0.668 | 0.150 | 0.706 | 0.554 |
| 22h | 0.663 | 12.913 | 0.636 | 0.613 | 0.681 | 0.122 | 0.668 | 0.598 |
| 23h | 0.660 | 19.896 | 0.783 | 0.489 | 0.676 | 0.113 | 0.628 | 0.659 |
| 24h | 0.671 | 15.195 | 0.704 | 0.577 | 0.687 | 0.100 | 0.575 | 0.717 |
